# Effects of Chinese herbal medicine on colorectal adenoma recurrence following polypectomy: a systematic review and meta-analysis

**DOI:** 10.3389/fphar.2025.1460900

**Published:** 2025-03-20

**Authors:** Yi Cheng, Yuan Ming Di, Brian May, Anthony Lin Zhang, Charlie Changli Xue, Beiping Zhang

**Affiliations:** ^1^ The China-Australia International Research Centre for Chinese Medicine, School of Health and Biomedical Sciences, STEM College, RMIT University, Melbourne, VIC, Australia; ^2^ Guangdong Provincial Hospital of Chinese Medicine, The Second Affiliated Hospital of Guangzhou University of Chinese Medicine, Guangdong Provincial Academy of Chinese Medical Sciences, Guangzhou, Guangdong, China; ^3^ State Key Laboratory of Dampness Syndrome of Chinese Medicine, The Second Affiliated Hospital of Guangzhou University of Chinese Medicine, Guangzhou, Guangdong, China

**Keywords:** Chinese herbal medicine, colorectal adenoma recurrence, *San zi* granule, *Si jun zi* decoction, systematic review, meta-analysis, colorectal cancer, colorectal polyp

## Abstract

**Objective:**

Preventing colorectal adenoma (CRA) recurrence after polypectomy is essential. However, the current evidence of Chinese herbal medicine (CHM) for CRA recurrence is still limited. This study aims to synthesize the effects of CHM as a prevention method for CRA recurrence.

**Methods:**

Nine databases were searched up to May 2024. Randomised controlled trials identifying the preventive effects of CHM among people with CRA post-polypectomy were included. spreadsheets were used to collect and extract data. RevMan and STATA were used for data analysis. We performed subgroup and sensitivity analyses to explore potentially influencing variables.

**Results:**

Twenty trials (2,325 participants) were included. The commonly used botanical drugs belonged to the categories of strengthening the spleen and anti-tumour metabolites. Compared to routine care (RC) alone, oral CHM plus RC significantly reduced the CRA recurrence rate at 12 months (RR 0.51, 95% CI [0.39, 0.67], I^2^ = 42%), 6 months (RR 0.44, 95% CI [0.36, 0.55], I^2^ = 0%), and 3 months (RR 0.46, 95% CI [0.22, 0.96], I^2^ = 0%) post-polypectomy. Compared to CHM placebo plus RC, *San zi* granule combined with RC significantly reduced CRA recurrence at 12 months post-polypectomy (RR 0.39, 95% CI [0.16, 0.93], I^2^ = 0%) and during the 2-year follow-up (RR 0.73, 95% CI [0.58, 0.90]). There were no significant differences between groups for treatment duration and syndromes. Additional analysis showed that oral CHM containing the botanical drugs of *Si jun zi* decoction plus RC reduced CRA recurrence at 12 months post-polypectomy with a low heterogeneity, compared to RC alone (RR 0.26, 95% CI [0.13, 0.54], I^2^ = 0%). Adverse events were similar in the above two comparisons.

**Conclusion:**

Oral CHM combined with RC may reduce CRA recurrence and be well-tolerated. *San zi* granule and *Si jun zi* decoction may be representative prescriptions Experimental studies of the frequent botanical drugs have found anti-cancer effects that may account for the clinical findings. Future rigorous clinical trials are needed due to low-to-moderate certainty of evidence.

**Systematic Review Registration:**

PROSPERO (CRD42023324197), https://www.crd.york.ac.uk/PROSPERO/view/CRD42023324197.

## 1 Introduction

Colorectal cancer (CRC) is regarded as the third most common malignancy worldwide, based on estimates from the World Health Organization in 2020 (Morgan et al., 2023). Approximately 70%–80% of CRCs develop from colorectal adenomas (CRA) through the adenoma–carcinoma pathway (Sung et al., 2022). CRA is a benign tumour in the large intestine and includes tubular, tubulovillous, villous, and serrated adenomas (Tanaka et al., 2020).

Since CRA plays an essential role in the development of CRC, polypectomy is the recommended primary treatment (Lieberman et al., 2012; Ferlitsch et al., 2017). However, CRA recurrence is common, varying from 19.3% to 59.46% at 1-year post-polypectomy (Yamaji et al., 2004; Gao et al., 2010; Shi et al., 2017). This rises to 87% at later follow-up times (>5 years) (Gao et al., 2010). Recurrence is associated with the number, size, and pathological results of adenomas diagnosed in the initial colonoscopy (Neugut et al., 1995; Huang et al., 2010). After polypectomy, people are treated with routine care (RC), consisting of fasting with energy support, liquid diet, or semi-liquid diet, as well as prevention for delayed bleeding (Association and Association, 2014). The duration of RC depends on the postoperative condition and usually lasts three to 7 days. The recommended follow-up interval times of repeated colonoscopy for people with adenoma range from one to 3 years, according to the condition of the lesion (number, size, and pathological type) (Colorectal Group, 2023). Currently, there is no established strategy for preventing CRA. Available pharmacotherapies have shown safety issues in clinical practice. Nonsteroidal anti-inflammatory drugs may cause gastrointestinal bleeding, especially in older people (Mahady et al., 2021), while non-selective cyclooxygenase-2 inhibitors may increase cardiovascular disease risk (Ribeiro et al., 2022). Therefore, new medications are being explored for CRA recurrence prevention. A meta-analysis with three trials and 1,076 participants revealed that berberine reduced the CRA recurrence rate, although there were more adverse events compared to the placebo (Fang et al., 2022). Another medication, metformin, was safe and effective for reducing the recurrence of colorectal polyps (including adenomas) at a low dose for 1 year of administration (Higurashi et al., 2016).

The effects of Chinese medicine (CM) on CRA recurrence have also been investigated. Two meta-analyses showed that CHM reduced the recurrence rate of colorectal polyps (including CRA) but did not focus on adenomas (Chen S. et al., 2020; Zhou et al., 2020). Another meta-analysis provided evidence on CHM for CRA and colorectal polyp recurrence. The primary outcome in this study was defined as the proportion of recurrent colorectal polyps instead of recurrent adenomas, which cannot reflect adenoma recurrence (Lin et al., 2020). Therefore, a systematic evaluation of the role of CHM on CRA recurrence is needed.

To address the above limitations, we conducted a systematic review to identify the clinical effects of CHM in preventing CRA recurrence and determine which botanical drugs and/or formulae are effective and safe.

## 2 Methods

We registered a protocol with PROSPERO (CRD42023324197) that was published (Cheng et al., 2023). This review was conducted with reference to the Cochrane Handbook (Higgins et al., 2022) and reported in accordance with Preferred Reporting Items for Systematic Reviews and Meta-Analyses (PRISMA) (Page et al., 2021).

### 2.1 Database and search strategy

We conducted a comprehensive search until May 2024 through five English databases (MEDLINE [via PubMed], Cochrane Library, EMBASE [via Embase.com], The Allied and Complementary Medicine Database [AMED], and Cumulative Index to Nursing and Allied Health Literature [CINAHL]) and four Chinese databases (Chinese Biology Medicine disc, China National Knowledge Infrastructure, China Science and Technology Journal Database, and Wanfang Data). No restrictions were applied. The search strategy combined medical terms and keywords relating to CRA, CHM, and randomised controlled trial (RCT) (Supplementary Appendix S10).

### 2.2 Inclusion and exclusion criteria

a. Study type: RCTs.
b. Participants: people aged 18 years or older with a pathological diagnosis of CRA (tubular, villous or tubulovillous (Tanaka et al., 2020)) and who had received a polypectomy to remove the colorectal lesions.
c. Intervention: use of CHM alone or combined with other therapies.
d. Comparators: RC (defined as the usual care after polypectomy to prevent bleeding and other postoperative complications (Association and Association, 2014), including fasting with energy support, liquid diet, or semi-liquid diet), CHM placebo, no additional treatment, or conventional medications.
e. Primary outcome: CRA recurrence rate in the repeated colonoscopy at any time point (Chen Y.-X. et al., 2020).
f. Secondary outcomes: a. number of people who had one or two CRAs, b. number of people who had three or more than three CRAs, c. number of people who had CRA with 1 cm size or larger, d. number of people who had advanced CRAs, e., number of people who were newly diagnosed with CRC, and f. adverse events (AEs).

We excluded studies that used the purified metabolites of CHM as interventions and/or did not provide specific data on the CRA recurrence rate. Trials were also excluded if CHM was used in the control group.

### 2.3 Data selection and extraction

Search results were checked, and duplicates were removed. Two reviewers (YC and YMD) conducted an initial selection of titles and abstracts, followed by an assessment of full texts for eligibility independently. The study data related to participants, interventions, comparators, and outcomes were extracted independently and double-checked by YC and YMD using a standardised spreadsheet. Any differences in selection and extraction were discussed with a third reviewer (ALZ). Authors were contacted by email if there were critical missing data.

### 2.4 Risk of bias assessment in included studies

Included studies were assessed by two reviewers (YC and YMD) independently using the Cochrane Collaboration Risk of Bias 2 (RoB 2) tool (Sterne et al., 2019). The following aspects were evaluated: randomisation procedure, intended interventions, missing outcome data, outcome measurement, and selection of the reported result (Sterne et al., 2019). Any disagreements were resolved by a senior reviewer (ALZ).

### 2.5 Statistical analysis

A narrative description was given when only one study was included in a comparison group. For comparison groups with more than one study, analyses were performed in Review Manager 5.4 (RevMan 5.4) and STATA (version 17.0) and presented with forest plots.

We used a risk ratio (RR) with a 95% confidence interval (CI) to assess dichotomous outcomes (CRA recurrence rate and the secondary outcomes) and risk difference (RD) to assess the absolute effect.

Considering the likely heterogeneity between the included studies, we selected the random-effects model for meta-analysis. Higgin’s I^2^ statistics were reported to assess heterogeneity between studies. We regarded an I^2^ value of more than 50% as substantial heterogeneity.

We conducted additional analyses based on the same CM formulae, treatment duration, comparator, and CM syndrome to explore the heterogeneity source and intervention effects.. We also performed a sensitivity analysis to evaluate the stability of results by omitting any one included trial and grouping the included studies based on different characteristics. Publication bias was measured by a funnel plot and Egger’s test if we included 10 or more studies.

### 2.6 Certainty of evidence

The overall certainty of evidence was evaluated by Grading of Recommendations, Assessment, Development and Evaluations (GRADE) (Guyatt et al., 2008). We rated the certainty of evidence on clinically significant outcomes (CRA recurrence rate and AEs).

## 3 Results

### 3.1 Characteristics of included studies

**FIGURE 1:**
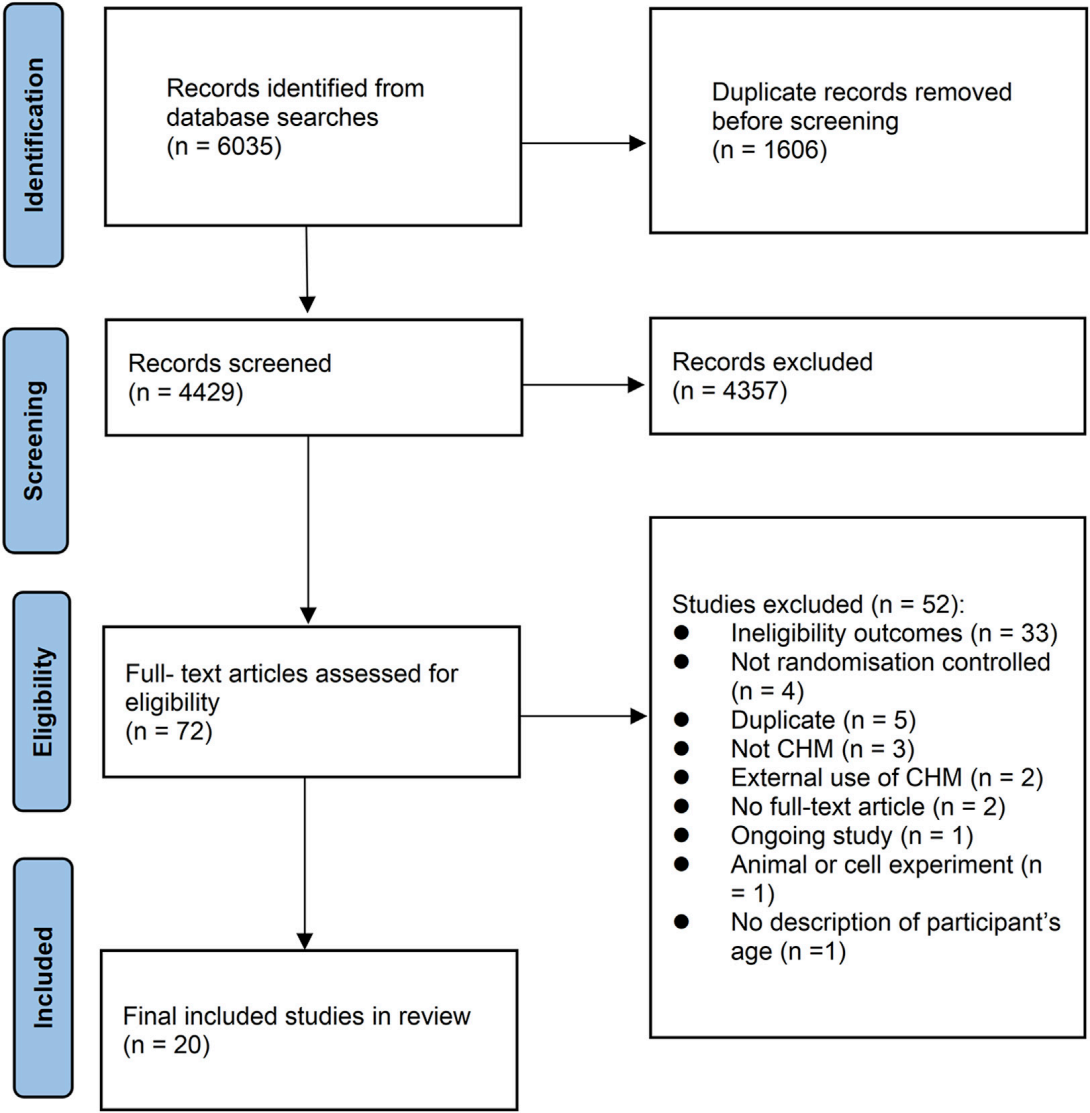
Study selection process.

A total of 6,035 citations were identified from the database search, and 4,429 titles and abstracts were screened after removing duplicate records. We assessed 72 full-text citations for eligibility, and 20 RCTs were included in the systematic review (Figure 1).

Five theses (Yuan, 2017; Xv, 2019; Zhao, 2020; Ren, 2021; Wang, 2022), one conference paper (Yan et al., 2018), and 14 published articles (Huang et al., 2018; Li L. et al., 2020; Xiong and Gu, 2020; Zhang et al., 2020; Li Y. et al., 2021; Pan, 2021; Xv et al., 2021; Li et al., 2022; Chen et al., 2023; Jia et al., 2023; Li C. et al., 2023; Li M. et al., 2023; Yao et al., 2023; Ni et al., 2024) were included. Study characteristics of included trials are summarised in Table 1.

**TABLE 1:** Characteristics of included studies.

Author, year	Article type	Number of cases (I/C)	Age (I) *mean ± SD*	Age (C) *mean ± SD*	Oral CHM treatment	Treatment time	Comparator	Outcomes	Time point for colonoscopy after polypectomy
Chen et al. (2023)	Journal article	67 (34/33)	39.87 ± 5.20	39.83 ± 5.23	*San zi* granule, 200 mL/day, bid	12 weeks	Routine care^a^ + CHM placebo, 200 mL/day, bid	RR, SA, CMCSS, OER, QoL, and CoRA	12 months
Huang et al. (2018)	Journal article	88 (44/44)	56.83 ± 9.29	57.74 ± 8.39	Modified *Li qi liu jun zi* decoction, bid	NS	Routine care^a^	RR, CMCSS, and OER	12 months
Jia et al. (2023)	Journal article	105 (53/52)	54.18 ± 4.39	53.85 ± 4.64	Modified *Yi qi san jie* formula, 400 mL/day, bid	8 weeks	Routine care^a^	RR, SA, CSSS, TM, and CoRA	3 months and 6 months
Li et al. (2023a)	Journal article	81 (42/39)	57.12 ± 10.69	58.17 ± 9.21	Modified *Wu mei* pill (NS)	12 weeks	Routine care^a^ + *Clostridium* butyricum capsule	RR, CSSS, and CMCSS	12 months
Li et al. (2023b)	Journal article	60 (30/30)	56.8 ± 10.6	54.8 ± 9.6	*Tian ma* granule, 1 bag/time, tid^b^	9 months	Routine care^a^	RR and CSSS	6 months and 12 months
Li et al. (2022)	Journal article	60 (30/30)	55.6	53.8	*San zi* granule, bid	3 months	Routine care^a^ + CHM placebo, bid	RR and CSSS	6 months
Li et al. (2021a)	Journal article	84 (42/42)	55. 98 ± 10. 48	53. 55 ± 11. 09	*Tiao chang xiao liu* formula, bid	3 months	Routine care^a^	RR, Beclin1, COX-2, p53, and SA	12 months
Li et al. (2020a)	Journal article	53 (30/23)	58.3 ± 6.0	58.1 ± 6.1	Modified *Jian pi xing qi* decoction, 400 mL/day, bid	3 months	Routine care^a^	RR, CMCSS, and QoL	6 months
Ni et al. (2024)	Journal article	336 (167/169)	55.4 ± 9.8	55.6 ± 10	*Shen bai* granule, bid	6 months^c^	Routine care^a^ + CHM placebo	RR, DRPL, and SA	12 months and 24 months
Pan (2021)	Journal article	103 (52/51)	65.23 ± 6.74	64.59 ± 6.68	Modified *Li qi liu jun zi* decoction, 400 mL/day, bid	8 weeks	Routine care^a^	RR, CMCSS, G-17, and OER	6 months
Ren (2021)	Thesis	58 (28/30)	NS	NS	Modified *Chu tan jie du* decoction, 100–150 mL/time, bid	2 months	Routine care^a^	RR, CMCSS, TM, FOBT, and CoRA	12 months
Wang (2022)	Thesis	58 (28/30)	NS	NS	*Sheng wan* paste, 30 g, bid	3 months	Routine care^a^	RR, SA, and CMCSS	12 months
Xiong and Gu (2020)	Journal article	120 (60/60)	48.2	49.5	Modified *Jian pi qv shi* formula, 400 mL/day, bid	3 months	Routine care^a^	RR and CSSS	6 months
Xv et al. (2021)	Journal article	96 (48/48)	53.5 ± 4.6	54.2 ± 4.4	Modified *Jian pi hua zhuo* formula, 200 mL/day, bid	3 months	Routine care^a^	RR, IL-6, COX-2, and CRP	3 months, 6 months, 12 months
Xv (2019)	Thesis	57 (28/29)	56 ± 12.97	55 ± 12.29	*Shen shao* paste, 20 mL/time, bid	3 months	Routine care^a^	RR, SA, and CMCSS	12 months
Yan et al. (2018)	Conference paper	167 (82/85)	55	55	*Zhu shao* granule (NS)	6 weeks	Routine care^a^	RR	6 months
Yao et al. (2023)	Journal article	80 (40/40)	56. 77 ± 9. 75	60. 63 ± 8. 10	*Yi qi hua shi xiao yu* formula, bid	12 weeks	Routine care^a^	RR, CMCSS, CoRA, LL, and OER	12 months
Yuan (2017)	Thesis	60 (30/30)	57.5 ± 11.5	56.8 ± 10.3	Modified *Wu mei* pill, 100 mL, bid	12 weeks	Routine care^a^	RR, SA, CSSS, and OER	6 months
Zhang et al. (2020)	Journal article	359 (176/183)	NS	NS	*Tiao chang xiao liu* formula, bid	3 months	Routine care^a^	RR and CMCSS	12 months
Zhao (2020)	Thesis	80 (40/40)	51.08 ± 5.70	50.38 ± 5.33	*Chang xi hua ji* decoction, 200 mL/time, bid	8 weeks	Routine care^a^	RR, SA, CMCSS, and OER	12 months

Abbreviations: C, control group; CHM, Chinese herbal medicine; CMCSS, Chinese medicine clinical syndrome score; CoRA, characteristics of recurrent adenomas (location, number, size, and pathology); COX-2, cyclooxygenase-2; CRP, C-reactive protein; CSSS, clinical symptom scale score; DRPL: detection rate of polypoid lesions (any polypoid lesions, high-risk adenoma, and sessile serrated lesions); FOBT, faecal occult blood test; G-17, Gastrin 17; I, intervention group; IL-6, interleukin-6; IL-17, interleukin-17; LL, lipid level; NS, not specified; OER, overall effective rate; QoL, quality of life; RR, recurrence rate of colorectal adenoma, SA, safety assessment; TM, tumour marker.

^a^ a Routine care contains energy support, fasting, and prevention for bleeding, usually remaining for 2–5 days after polypectomy, and health education during the follow-up.

^b^ The treatment cycle was 1 year, and the drug was taken for 3 months and stopped for 1 month, accounting to four months for a course of treatment.

^c^ The drug was taken for 3 months as a course. This drug was given as one course in the first year of enrolment and one course in the initial 3 months of the second year.

RCTs were conducted in China and included 2,325 participants (1,165 in treatment groups and 1,160 in control groups) aged 18–79. All participants were diagnosed with CRA and underwent a polypectomy to remove the CRA completely.

All included RCTs used oral CHM combined with RC as the intervention. Treatment duration ranged from 6 weeks to 9 months, except that one study did not clarify the exact treatment duration (Huang et al., 2018). Thirteen out of 20 studies reported CM syndrome differentiation. Eleven studies treated participants depending on syndrome differentiation.

Sixteen studies compared oral CHM plus RC with RC (Yuan, 2017; Huang et al., 2018; Yan et al., 2018; Xv, 2019; Li L. et al., 2020; Xiong and Gu, 2020; Zhang et al., 2020; Zhao, 2020; Li Y. et al., 2021; Pan, 2021; Ren, 2021; Xv et al., 2021; Wang, 2022; Jia et al., 2023; Li M. et al., 2023; Yao et al., 2023), three compared oral CHM plus RC with CHM placebo plus RC (Li et al., 2022; Chen et al., 2023; Ni et al., 2024), and one compared oral CHM plus RC with the probiotic (*Clostridium butyricum* capsule) plus RC (Li C. et al., 2023). All RCTs reported on the CRA recurrence rate diagnosed by colonoscopy and biopsy, nine studies evaluated CRA recurrence rate at 6 months (Yuan, 2017; Yan et al., 2018; Li L. et al., 2020; Xiong and Gu, 2020; Pan, 2021; Xv et al., 2021; Li et al., 2022; Jia et al., 2023; Li M. et al., 2023), 13 studies had an assessment of CRA recurrence at 12 months (Huang et al., 2018; Xv, 2019; Zhang et al., 2020; Zhao, 2020; Li Y. et al., 2021; Ren, 2021; Xv et al., 2021; Li et al., 2022; Wang, 2022; Chen et al., 2023; Li C. et al., 2023; Li M. et al., 2023; Yao et al., 2023), and one assessed recurrence rate during a 2-year follow-up (proportion of participants with at least one recurrent adenoma in any repeated colonoscopies during the follow-up). Twelve trials assessed and reported on AEs (Yuan, 2017; Xv, 2019; Li L. et al., 2020; Zhang et al., 2020; Zhao, 2020; Li Y. et al., 2021; Ren, 2021; Xv et al., 2021; Wang, 2022; Chen et al., 2023; Jia et al., 2023; Ni et al., 2024), and two studies included a new diagnosis of CRC as an outcome (Xv, 2019; Chen et al., 2023). Two included studies (Zhang et al., 2020; Ni et al., 2024) assessed the number of recurrent advanced CRAs, and one study (Yao et al., 2023) reported recurrent adenomas sized 1 cm or larger.

### 3.2 Summary of CHM use

Modified *Li qi liu jun zi* decoction (Huang et al., 2018; Pan, 2021), *San zi* granule (Li et al., 2022; Chen et al., 2023), *Tiao chang xiao liu* formula (Zhang et al., 2020; Li Y. et al., 2021), and modified *Wu mei* pill (Yuan, 2017; Li C. et al., 2023) were each used as an intervention in two studies. The remaining 12 studies used different formulae. The formula names in Chinese *Pin yin*, sources, botanical drugs, and quality control information are provided in Table 2.

**TABLE 2:** Information about formulae.

Study, year	Formula name	Source	Composition and dosage
Chen et al. (2023)	*San zi* granule	Jiangyin Tianjiang Pharmaceutical Co., Ltd	*Astragalus mongholicus* Bunge [Fabaceae; Astragali radix] (*Huang qi*) 10 g *Prunus mume* (Sieb.) Sieb.et Zucc. [Rosaceae; Mume fructus] (*Wu mei*) 10 g *Punica granatum* L. [Lythraceae; Semen punicae granati] (*Shi liu zi*) 10 g *Entada phaseoloides* (Linn.) Merr. [Fabaceae; Entadae semen] (*Ke teng zi*) 10 g *Phyllanthus emblica* L. [Phyllanthaceae; Phyllanthi fructus] (*Yu gan zi*) 10 g *Zanthoxylum schinifolium* Siebold & Zucc. [Rutaceae; Zanthoxyli pericarpium] (*Hua jiao*) 3 g *Uncaria rhynchophylla* (Miq.) Miq. [Rubiaceae; Uncariae ramulus cum uncis] (*Gou teng*) 10 g *Curcuma wenyujin* Y. H. Chen et C. Ling [Zingiberaceae; Curcumae radix] (*Yu jin*) 10 g *Glycyrrhiza uralensis* Fisch. ex DC. [Fabaceae; Glycyrrhizae radix et rhizoma] (*Gan cao*) 6 g
Huang et al. (2018)	Modified *Li qi liu jun zi* decoction	NI	*Codonopsis pilosula* (Franch.) Nannf. [Campanulaceae; Codonopsis radix] (*Dang shen*) 20 g *Poria cocos* (Schw.) Wolf [Polyporaceae; Poria] (*Fu ling*) 15 g *Atractylodes macrocephala* Koidz. [Asteraceae; Atractylodis macrocephalae rhizoma] (*Bai zhu*) 15 g *Paeonia lactiflora* Pall. [Paeoniaceae; Paeoniae radix alba] (*Bai shao*) 15 g *Bupleurum chinense* DC. [Apiaceae; Bupleuri radix] (*Chai hu*) 10 g *Citrus reticulata* Blanco [Rutaceae; Citri reticulatae pericarpium] (*Chen pi*) 5 g *Panax notoginseng* (Burkill) F.H.Chen [Araliaceae; Notoginseng radix et rhizoma] (*San qi*) 5 g *Magnolia officinalis* Rehder & E.H.Wilson [Magnoliaceae; Magnoliae officinalis flos] (*Hou po hua*) 6 g *Pinellia ternata* (Thunb.) Makino [Araceae; Pinelliae rhizoma] (*Ban xia*) 12 g *Citrus × aurantium* L. [Rutaceae; Aurantii fructus] (*Zhi ke*) 10 g *Salvia miltiorrhiza* Bunge [Lamiaceae; Salviae miltiorrhizae radix et rhizoma] (*Dan shen*) 10 g *Glycyrrhiza uralensis* Fisch. ex DC. [Fabaceae; Glycyrrhizae radix et rhizoma] (*Gan cao*) 5 g
Jia et al. (2023)	Modified *Yi qi san jie* formula	NI	*Agrimonia pilosa* Ledeb. [Rosaceae; Agrimoniae herba] (*Xian he cao*) 30 g *Poria cocos* (Schw.) Wolf [Polyporaceae; Poria] (*Fu ling*) 20 g *Codonopsis pilosula* (Franch.) Nannf. [Campanulaceae; Codonopsis radix] (*Dang shen*) 15 g *Astragalus mongholicus* Bunge [Fabaceae; Astragali radix] (*Huang qi*) 15 g *Atractylodes macrocephala* Koidz. [Asteraceae; Atractylodis macrocephalae rhizoma] (*Bai zhu*) 15 g *Patrinia villosa* (Thunb.) Dufr. [Caprifoliaceae; Patriniae herba] (*Bai jiang cao*) 15 g *Scleromitrion diffusum* (Willd.) R.J.Wang [Rubiaceae; Hedyotis diffusae herba] (*Bai hua she she cao*) 15 g *Scutellaria barbata* D.Don [Lamiaceae; Scutellariae barbatae herba] (*Ban zhi lian*) 15 g *Trionyx sinensis* Wiegmann [Trionychidae; Trionycis carapax] (*Bie jia*) 15 g *Ranunculus ternatus* Thunb. [Ranunculaceae; Ranunculi ternati radix] (*Mao zhao cao*) 15 g *Phytolacca acinosa* Roxb. [Phytolaccaceae; Phytolaccae radix] (*Shang lu*) 6 g *Eupatorium fortunei* Turcz. [Asteraceae; Eupatorii herba] (*Pei lan*) 6 g *Prunus mume* (Sieb.) Sieb.et Zucc. [Rosaceae; Mume fructus] (*Wu mei*) 6 g *Bombyx mori* Linnaeus. [Bombycidae; Bombyx batryticatus] (*Jiang can*) 6 g *Glycyrrhiza uralensis* Fisch. ex DC. [Fabaceae; Glycyrrhizae radix et rhizoma] (*Gan cao*) 5 g *Panax notoginseng* (Burkill) F.H.Chen [Araliaceae; Notoginseng radix et rhizoma] (*San qi*) 3 g
Li et al. (2023a)	Modified *Wu mei p*ill	Pharmacy of ShangHai GuangHua Hospital of Integrated Traditional Chinese and Western Medicine	*Prunus mume* (Sieb.) Sieb.et Zucc. [Rosaceae; Mume fructus] (*Wu mei*) 15 g *Pseudostellaria heterophylla* (Miq.) Pax [Caryophyllaceae; Pseudostellariae radix] (*Tai zi shen*) 10 g *Asarum heterotropoides* F.Schmidt [Aristolochiaceae; Asari radix et rhizoma] (*Xi xin*) 3 g *Cinnamomum cassia* (L.) J.Presl [Lauraceae; Cinnamomi ramulus] (*Gui zhi*) 6 g *Coptis chinensis* Franch. [Ranunculaceae; Coptidis rhizoma] (*Huang lian*) 3 g *Phellodendron chinense* C.K.Schneid. [Rutaceae; Phellodendri chinensis cortex] (*Huang bo*) 6 g *Angelica sinensis*(Oliv.)Diels [Apiaceae; Angelica sinensis (Oliv.) Diels] (*Dang gui*) 10 g *Aconitum carmichaelii* Debeaux [Ranunculaceae; Aconiti radix lateralis praeparata] (*Fu zi*) 3 g *Zingiber officinale* Roscoe [Zingiberaceae; Zingiberis rhizoma] (*Gan jiang*) 6 g
Li et al. (2023b)	*Tian ma* granule^a^	Pharmacy of Huanan Academy of Chinese medicine	* Scolopendra subspinipes mutilans L. Koch [Scolopendridae; Scolopendra] (Wu gong) NS*. Buthus martensii Karsch [Buthidae; Scorpio] (Quan xie) NS. Lobelia chinensis Lour. [Campanulaceae; Lobeliae chinensis herba] (Ban bian lian) NS. Phellodendron chinense C.K.Schneid. [Rutaceae; Phellodendri chinensis cortex] (Huang bo) NS. Sparganium stoloniferum (Buch.-Ham. ex Graebn.) Buch.-Ham. ex Juz. [Typhaceae; Sparganii rhizoma] (San leng) NS. Arisaema erubescens (Wall.) Schott [Araceae; Arisaema cum bile] (Dan nan xing) NS. Sargassum pallidum (Turn.) C.Ag. [Sargassaceae; Sargassum] (Hai zao) NS. Astragalus mongholicus Bunge [Fabaceae; Astragali radix] (Huang qi) NS. Dioscorea oppositifolia L. [Dioscoreaceae; Dioscoreae rhizoma] (Shan yao) NS. Rheum palmatum L. [Polygonaceae; Rhei radix et rhizoma] (Da huang) NS.
Li et al. (2022)	*San zi* granule	NI	*Astragalus mongholicus* Bunge [Fabaceae; Astragali radix] (*Huang qi*) 10 g *Prunus mume* (Sieb.) Sieb.et Zucc. [Rosaceae; Mume fructus] (*Wu mei*) 10 g *Punica granatum* L. [Lythraceae; Semen punicae granati] (*Shi liu zi*) 10 g *Entada phaseoloides* (Linn.) Merr. [Fabaceae; Entadae semen] (*Ke teng zi*) 10 g *Phyllanthus emblica* L. [Phyllanthaceae; Phyllanthi fructus] (*Yu gan zi*) 10 g *Zanthoxylum schinifolium* Siebold & Zucc. [Rutaceae; Zanthoxyli pericarpium] (*Hua jiao*) 3 g *Uncaria rhynchophylla* (Miq.) Miq. [Rubiaceae; Uncariae ramulus cum uncis] (*Gou teng*) 10 g *Curcuma wenyujin* Y. H. Chen et C. Ling [Zingiberaceae; Curcumae radix] (*Yu jin*) 10 g *Glycyrrhiza uralensis* Fisch. ex DC. [Fabaceae; Glycyrrhizae radix et rhizoma] (*Gan cao*) 6 g
Li et al. (2021b)	*Tiao chang xiao liu* formula	Pharmacy of Guangdong Provincial Hospital of Chinese Medicine	*Codonopsis pilosula* (Franch.) Nannf. [Campanulaceae; Codonopsis radix] (*Dang shen*) 15 g *Atractylodes macrocephala* Koidz. [Asteraceae; Atractylodis macrocephalae rhizoma] (*Bai zhu*) 15 g *Astragalus mongholicus* Bunge [Fabaceae; Astragali radix] (*Huang qi*) 15 g *Coix lacryma-jobi* L.var.*ma-yuen* (Roman.) Stapf [Poaceae; Coicis semen] (*Yi yi ren*) 30 g *Amomum Krervanh* Pierre ex Gagnep. [Zingiberaceae; Amomi fructus rotundus] (*Dou kou*) 10 g *Scleromitrion diffusum* (Willd.) R.J.Wang [Rubiaceae; Hedyotis diffusae herba] (*Bai hua she she cao*) 30 g *Curcuma phaeocaulis* Valeton [Zingiberaceae; Curcumae rhizoma] (*E zhu*) 15 g *Panax notoginseng (Burkill) F.H.Chen [Araliaceae; Notoginseng radix et rhizoma]* (*San qi*) 10 g *Glycyrrhiza uralensis* Fisch. ex DC. [Fabaceae; Glycyrrhizae radix et rhizoma] (*Gan cao*) 5 g
Li et al. (2020b)	Modified *Jian pi xing qi* decoction	NI	*Salvia chinensis* Benth. [Lamiaceae; Salviae chinensis herba] (*Shi jian chuan*) 25 g *Coix lacryma-jobi* L.var.*ma-yuen* (Roman.) Stapf [Poaceae; Coicis semen] (*Yi yi ren*) 25 g *Dioscorea oppositifolia* L. [Dioscoreaceae; Dioscoreae rhizoma] (*Shan yao*) 20 g *Poria cocos* (Schw.) Wolf [Polyporaceae; Poria] (*Fu ling*) 15 g *Codonopsis pilosula* (Franch.) Nannf. [Campanulaceae; Codonopsis radix] (*Dang shen*) 15 g *Crataegus pinnatifida* Bunge [Rosaceae; Crataegi fructus] (*Shan zha*) 15 g *Prunus mume* (Sieb.) Sieb. et Zucc. [Rosaceae; Mume fructus] (*Wu mei*) 10 g *Curcuma phaeocaulis* Valeton [Zingiberaceae; Curcumae rhizoma] (*E zhu*) 10 g *Atractylodes macrocephala* Koidz. [Asteraceae; Atractylodis macrocephalae rhizoma] (*Bai zhu*) 10 g *Aucklandia lappa* (Decne.) Decne. [Asteraceae; Aucklandiae radix] (*Mu xiang*) 10 g *Glycyrrhiza uralensis* Fisch. ex DC. [Fabaceae; Glycyrrhizae radix et rhizoma] (*Gan cao*) 6 g *Citrus reticulata* Blanco [Rutaceae; Citri reticulatae pericarpium] (*Chen pi*) 6 g
Ni et al. (2024)	*Shen bai* granule	Jiangyin Tianjiang Pharmaceutical Co., LTD.	*Sophora flavescens* Aiton [Fabaceae; Sophorae flavescentis radix] (*Ku shen*) 4.5 g *Scleromitrion diffusum* (Willd.) R.J.Wang [Rubiaceae; Hedyotis diffusae herba] (*Bai hua she she cao*) 10 g *Codonopsis pilosula* (Franch.) Nannf. [Campanulaceae; Codonopsis radix] (*Dang shen*) 7.5 g *Atractylodes macrocephala* Koidz. [Asteraceae; Atractylodis macrocephalae rhizoma] (*Bai zhu*) 6 g *Coix lacryma-jobi* L.var.*ma-yuen* (Roman.) Stapf [Poaceae; Coicis semen] (*Yi yi ren*) 10 g *Coptis chinensis* Franch. [Ranunculaceae; Coptidis rhizoma] (*Huang lian*) 1.5 g *Prunus mume* (Sieb.) Sieb.et Zucc. [Rosaceae; Mume fructus] (*Wu mei*) 4.5 g *Zingiber officinale* Roscoe [Zingiberaceae; Zingiberis rhizoma praeparatum] (*Pao jiang*) 3 g
Pan (2021)	Modified *Li qi liu jun zi* decoction	NI	*Codonopsis pilosula* (Franch.) Nannf. [Campanulaceae; Codonopsis radix] (*Dang shen*) 20 g *Poria cocos* (Schw.) Wolf [Polyporaceae; Poria] (*Fu ling*) 15 g *Atractylodes macrocephala* Koidz. [Asteraceae; Atractylodis macrocephalae rhizoma] (*Bai zhu*) 15 g *Paeonia lactiflora* Pall. [Paeoniaceae; Paeoniae radix alba] (*Bai shao*) 15 g *Bupleurum chinense* DC. [Apiaceae; Bupleuri radix] (*Chai hu*) 10 g *Citrus reticulata* Blanco [Rutaceae; Citri reticulatae pericarpium] (*Chen pi*) 5 g *Panax notoginseng* (Burkill) F.H.Chen [Araliaceae; Notoginseng radix et rhizoma] (*San qi*) 5 g *Magnolia officinalis* Rehder & E.H.Wilson [Magnoliaceae; Magnoliae officinalis flos] (*Hou po hua*) 6 g *Pinellia ternata* (Thunb.) Makino [Araceae; Pinelliae rhizoma] (*Ban xia*) 12 g *Citrus × aurantium* L. [Rutaceae; Aurantii fructus] (*Zhi ke*) 10 g *Salvia miltiorrhiza* Bunge [Lamiaceae; Salviae miltiorrhizae radix et rhizoma] (*Dan shen*) 10 g *Glycyrrhiza uralensis* Fisch. ex DC. [Fabaceae; Glycyrrhizae radix et rhizoma] (*Gan cao*) 5 g
Ren (2021)	Modified *Chu tan jie du* decoction	Kangmei Pharmaceutical Co., Ltd	*Aucklandia lappa* (Decne.) Decne. [Asteraceae; Aucklandiae radix] (*Mu xiang*) 7 g *Amomum villosum* Lour. [Zingiberaceae; Amomi fructus] (*Sha ren*) 7 g *Codonopsis pilosula* (Franch.) Nannf. [Campanulaceae; Codonopsis radix] (*Dang shen*) 15f *Atractylodes macrocephala* Koidz. [Asteraceae; Atractylodis macrocephalae rhizoma] (*Bai zhu*) 9 g *Coix lacryma-jobi L. var. ma-yuen.ma-yuen* (Roman.) Stapf [Poaceae; Coicis semen] (*Yi yi ren*) 50 g *Citrus reticulata* Blanco [Rutaceae; Citri reticulatae pericarpium] (*Chen pi*) 5 g *Poria cocos* (Schw.) Wolf [Polyporaceae; Poria] (*Fu ling*) 15 g *Taxus chinensis* (Pilg.) Rehder [Taxaceae; Taxus chinensis] (*Hong dou shan*) 3 g *Magnolia officinalis* Rehder & E.H.Wilson [Magnoliaceae; Magnoliae officinalis cortex] (*Hou po*) 10 g Pinellia ternata (Thunb.) Makino [Araceae; Pinelliae rhizoma] (*Ban xia*) 5 g *Curcuma phaeocaulis* Valeton [Zingiberaceae; Curcumae rhizoma] (*E zhu*) 5 g *Sparganium stoloniferum* (Buch.-Ham. ex Graebn.) Buch.-Ham. ex Juz. [Typhaceae; Sparganii rhizoma] (*San leng*) 5 g *Clematis chinensis* Osbeck [Ranunculaceae; Clematidis radix et rhizoma] (*Wei ling xian*) 7 g *Houttuynia cordata* Thunb. [Saururaceae; Houttuyniae herba] (*Yu xing cao*) 15 g *Agrimonia pilosa* Ledeb. [Rosaceae; Agrimoniae herba] (*Xian he cao*) 15 g *Fritillaria thunbergii* Miq. [Liliaceae; Fritillariae thunbergii bulbus] (*Zhe bei mu*) 15 g *Fagopyrum dibotrys* (D.Don) Hara [Polygonaceae; Fagopyri dibotryis rhizoma] (*Jin qiao mai*) 15 g
Wang (2022)	*Sheng wan* paste^a^	Affiliated Hospital of Shandong University of Traditional Chinese Medicine	*Codonopsis pilosula* (Franch.) Nannf. [Campanulaceae; Codonopsis radix] (*Dang shen*) *Atractylodes macrocephala* Koidz. [Asteraceae; Atractylodis macrocephalae rhizoma] (*Bai zhu*) *Poria cocos* (Schw.) Wolf [Polyporaceae; Poria] (*Fu ling*) *Glycyrrhiza uralensis* Fisch. ex DC. [Fabaceae; Glycyrrhizae radix et rhizoma] (*Gan cao*) *Astragalus mongholicus* Bunge [Fabaceae; Astragali radix] (*Huang qi*) *Sparganium stoloniferum* (Buch.-Ham. ex Graebn.) Buch.-Ham. ex Juz. [Typhaceae; Sparganii rhizoma] (*San leng*) *Curcuma phaeocaulis* Valeton [Zingiberaceae; Curcumae rhizoma] (*E zhu*) *Salvia miltiorrhiza* Bunge [Lamiaceae; Salviae miltiorrhizae radix et rhizoma] (*Dan shen*) *Scutellaria barbata* D.Don [Lamiaceae; Scutellariae barbatae herba] (*Ban zhi lian*) *Paris polyphylla* Sm. [Melanthiaceae; Paridis rhizoma] (*Chong lou*) *Terminalia chebula* Retz. [Combretaceae; Chebulae fructus] (*He zi*) *Taraxacum mongolicum* Hand. -Mazz. [Asteraceae; Taraxaci herba] (*Pu gong ying*) *Cremastra appendiculata* (D.Don) Makino [Orchidaceae; Cremastrae pseudobulbus] (*Shan ci gu*) *Lilium lancifolium* Thunb. [Liliaceae; Lilii bulbus] (*Bai he*) *Gentiana manshurica* Kitag. [Gentianaceae; Gentianae radix et rhizoma] (*Long dan*) *Cullen corylifolium* (L.) Medik. [Fabaceae; Psoraleae fructus] (*Bu gu zhi*)
Xiong and Gu (2020)	Modified *Jian pi qv shi* formula	NI	*Codonopsis pilosula* (Franch.) Nannf. [Campanulaceae; Codonopsis radix] (*Dang shen*) 15 g *Astragalus mongholicus* Bunge [Fabaceae; Astragali radix] (*Huang qi*) 30 g *Poria cocos* (Schw.) Wolf [Polyporaceae; Poria] (*Fu ling*) 10 g *Polyporus umbellatus* (Pers.) Fries [Polygonaceae; Polyporus] (*Zhu ling*) 10 g *Prunus mume* (Sieb.) Sieb.et Zucc. [Rosaceae; Mume fructus] (*Wu mei*) 30 g *Atractylodes lancea* (Thunb.) DC. [Asteraceae; Atractylodis rhizoma] (*Cang zhu*) 10 g *Scutellaria baicalensis* Georgi [Lamiaceae; Scutellariae radix] (*Huang qin*) 10 g *Paeonia veitchii* Lynch [Paeoniaceae; Paeoniae radix rubra] (*Chi shao*) 10 g *Dioscorea oppositifolia* L. [Dioscoreaceae; Dioscoreae rhizoma] (*Shan yao*) 15 g *Coix lacryma-jobi* L.var.*ma-yuen* (Roman.) Stapf [Poaceae; Coicis semen] (*Yi yi ren*) 15 g *Eupatorium fortunei* Turcz. [Asteraceae; Eupatorii herba] (*Pei lan*) 10 g *Crataegus pinnatifida* Bunge [Rosaceae; Crataegi fructus] (*Shan zha*) 15 g *Massa medicata* Fermentata [Medicated leaven] (*Jiao shen qu*) 15 g *Citrus reticulata* Blanco [Rutaceae; Citri reticulatae pericarpium] (*Chen pi*) 5 g *Glycyrrhiza uralensis* Fisch. ex DC. [Fabaceae; Glycyrrhizae radix et rhizoma] (*Gan cao*) 3 g
Xv et al. (2021)	Modified *Jian pi hua zhuo* formula	Pharmacy of Hubei Provincial Hospital of Traditional Chinese Medicine	*Prunus mume* (Sieb.) Sieb.et Zucc. [Rosaceae; Mume fructus] (*Wu mei*) 20 g *Curcuma longa* L. [Zingiberaceae; Curcumae longae rhizoma] (*Jiang huang*) 10 g *Coptis chinensis* Franch. [Ranunculaceae; Coptidis rhizoma] (*Huang lian*) 6 g *Astragalus mongholicus* Bunge [Fabaceae; Astragali radix] (*Huang qi*) 15 g *Codonopsis pilosula* (Franch.) Nannf. [Campanulaceae; Codonopsis radix] (*Dang shen*) 10 g *Atractylodes macrocephala* Koidz. [Asteraceae; Atractylodis macrocephalae rhizoma] (*Bai zhu*) 12 g *Poria cocos* (Schw.) Wolf [Polyporaceae; Poria] (*Fu ling*) 15 g *Coix lacryma-jobi* L.var.*ma-yuen* (Roman.) Stapf [Poaceae; Coicis semen] (*Yi yi ren*) 40 g *Citrus reticulata* Blanco [Rutaceae; Citri reticulatae pericarpium] (*Chen pi*) 12 g *Platycodon grandiflorum* (Jacq.) A.DC. [Campanulaceae; Platycodonis radix] (*Jie geng*) 10 g *Pinellia ternate* (Thunb.) Makino [Araceae; Pinelliae rhizoma] (*Ban xia*) 9 g *Bombyx mori* Linnaeus. [Bombycidae; Bombyx batryticatus] (*Jiang can*) 15 g *Agrimonia pilosa* Ledeb. [Rosaceae; Agrimoniae herba] (*Xian he cao*) 15 g *Scleromitrion diffusum* (Willd.) R.J.Wang [Rubiaceae; Hedyotis diffusae herba] (*Bai hua she she cao*) 20 g
Xv (2019)	*Shen shao* paste^b^	NI	*Codonopsis pilosula* (Franch.) Nannf. [Campanulaceae; Codonopsis radix] (*Dang shen*) 150 g *Atractylodes macrocephala* Koidz. [Asteraceae; Atractylodis macrocephalae rhizoma] (*Bai zhu*) 150 g *Poria cocos* (Schw.) Wolf [Polyporaceae; Poria] (*Fu ling*) 150 g *Cinnamomum cassia* (L.) J.Presl [Lauraceae; Cinnamomi ramulus] (*Gui zhi*) 150 g *Paeonia veitchii* Lynch [Paeoniaceae; Paeoniae radix rubra] (*Chi shao*) 150 g *Prunus persica* (L.) Batsch [Rosaceae; Persicae semen] (*Tao ren*) 120 g *Paeonia × suffruticosa* Andrews [Paeoniaceae; Moutan cortex] (*Mu dan pi*) 120 g *Citrus reticulata* Blanco [Rutaceae; Citri reticulatae pericarpium] (*Chen pi*) 120 g *Trionyx sinensis* Wiegmann [Trionychidae; Trionycis carapax] (*Bie jia*) 100 g *Paeonia lactiflora* Pall. [Paeoniaceae; Paeoniae radix alba] (*Bai shao*) 300 g *Saposhnikovia divaricata* (Turcz. ex Ledeb.) Schischk. [Apiaceae; Saposhnikoviae radix] (*Fang feng*) 120 g *Glycyrrhiza uralensis* Fisch. ex DC. [Fabaceae; Glycyrrhizae radix et rhizoma] (*Gan cao*) 60 g
Yan et al. (2018)	*Zhu shao* granule^a^	Yantai Hospital of Traditional Chinese Medicine	*Paeonia lactiflora* Pall. [Paeoniaceae; Paeoniae radix alba] (*Bai shao*) *Atractylodes macrocephala* Koidz. [Asteraceae; Atractylodis macrocephalae rhizoma] (*Bai zhu*) *Astragalus mongholicus* Bunge [Fabaceae; Astragali radix] (*Huang qi*) *Citrus reticulata* Blanco [Rutaceae; Citri reticulatae pericarpium] (*Chen pi*) *Dolichos lablab* L. [Fabaceae; Lablab semen album] (*Bai bian dou*) *Terminalia chebula* Retz. [Combretaceae; Chebulae fructus] (*He zi*) *Cullen corylifolium* (L.) Medik. [Fabaceae; Psoraleae fructus] (*Bu gu zhi*) *Saposhnikovia divaricata* (Turcz. ex Ledeb.) Schischk. [Apiaceae; Saposhnikoviae radix] (*Fang feng*) *Amomum villosum* Lour. [Zingiberaceae; Amomi fructus] (*Sha ren*) *Cinnamomum cassia* (L.) J.Presl [Lauraceae; Cinnamomi ramulus] (*Gui zhi*) *Ziziphus jujuba* Mill. [Rhamnaceae; Jujubae fructus] (*Da zao*) *Zingiber officinale* Roscoe [Zingiberaceae; Zingiberis rhizoma] (*Gan jiang*) *Lindera aggregata* (Sims) Kosterm. [Lauraceae; Linderae radix] (*Wu yao*) *Glycyrrhiza uralensis* Fisch. ex DC. [Fabaceae; Glycyrrhizae radix et rhizoma] (*Gan cao*)
Yao et al. (2023)	*Yi qi hua shi xiao yu* formula	Pharmacy of Nanjing University of Chinese Medicine Affiliated Nanjing Combination of Chinese Traditional and Western Medicine Hospital	*Astragalus mongholicus* Bunge [Fabaceae; Astragali radix] (*Huang qi*) 10 g *Prunus mume* (Sieb.) Sieb.et Zucc. [Rosaceae; Mume fructus] (*Wu mei*) 10 g *Citrus reticulata* Blanco [Rutaceae; Citri reticulatae pericarpium] (*Chen pi*) 6 g *Coix lacryma-jobi* L.var.*ma-yuen* (Roman.) Stapf [Poaceae; Coicis semen] (*Yi yi ren*) 15 g *Curcuma wenyujin* Y. H. Chen et C. Ling [Zingiberaceae; Curcumae radix] (*Yu jin*) 10 g *Zanthoxylum schinifolium* Siebold & Zucc. [Rutaceae; Zanthoxyli pericarpium] (*Hua jiao*) 3 g *Scleromitrion diffusum* (Willd.) R.J.Wang [Rubiaceae; Hedyotis diffusae herba] (*Bai hua she she cao*) 15 g *Glycyrrhiza uralensis* Fisch. ex DC. [Fabaceae; Glycyrrhizae radix et rhizoma] (*Gan cao*) 6 g
Yuan (2017)	Modified *Wu mei* pill	NI	*Prunus mume* (Sieb.) Sieb.et Zucc. [Rosaceae; Mume fructus] (*Wu mei*) 30 g *Pseudostellaria heterophylla* (Miq.) Pax [Caryophyllaceae; Pseudostellariae radix] (*Tai zi shen*) 10 g *Atractylodes macrocephala* Koidz. [Asteraceae; Atractylodis macrocephalae rhizoma] (*Bai zhu*) 10 g *Zingiber officinale* Roscoe [Zingiberaceae; Zingiberis rhizoma praeparatum] (*Pao jiang*) 5 g *Coptis chinensis* Franch. [Ranunculaceae; Coptidis rhizoma] (*Huang lian*) 3 g *Aucklandia lappa* (Decne.) Decne. [Asteraceae; Aucklandiae radix] (*Mu xiang*) 10 g *Citrus reticulata* Blanco [Rutaceae; Citri reticulatae pericarpium] (*Chen pi*) 10 g *Coix lacryma-jobi* L.var.*ma-yuen* (Roman.) Stapf [Poaceae; Coicis semen] (*Yi yi ren*) 20 g *Cimicifuga heracleifolia* Kom. [Ranunculaceae; Cimicifugae rhizoma] (*Sheng ma*) 10 g *Glycyrrhiza uralensis* Fisch. ex DC. [Fabaceae; Glycyrrhizae radix et rhizoma] (*Gan cao*) 3 g
Zhang et al. (2020)	*Tiao chang xiao liu* formula	Pharmacy of Guangdong Provincial Hospital of Chinese Medicine	*Codonopsis pilosula* (Franch.) Nannf. [Campanulaceae; Codonopsis radix] (*Dang shen*) 15 g *Atractylodes macrocephala* Koidz. [Asteraceae; Atractylodis macrocephalae rhizoma] (*Bai zhu*) 15 g *Astragalus mongholicus* Bunge [Fabaceae; Astragali radix] (*Huang qi*) 15 g *Coix lacryma-jobi* L.var.*ma-yuen* (Roman.) Stapf [Poaceae; Coicis semen] (*Yi yi ren*) 30 g *Amomum Krervanh* Pierre ex Gagnep. [Zingiberaceae; Amomi fructus rotundus] (*Dou kou*) 10 g *Scleromitrion diffusum* (Willd.) R.J.Wang [Rubiaceae; Hedyotis diffusae herba] (*Bai hua she she cao*) 30 g *Curcuma phaeocaulis* Valeton [Zingiberaceae; Curcumae rhizoma] (*E zhu*) 15 g *Panax notoginseng* (Burkill) F.H.Chen [Araliaceae; Notoginseng radix et rhizoma (*San qi*) 10 g *Glycyrrhiza uralensis* Fisch. ex DC. [Fabaceae; Glycyrrhizae radix et rhizoma] (*Gan cao*) 5 g
Zhao (2020)	*Chang xi hua ji* decoction	Pharmacy of Inner Mongolia Hospital of Traditional Chinese Medicine	*Astragalus mongholicus* Bunge [Fabaceae; Astragali radix] (*Huang qi*) 20 g *Codonopsis pilosula* (Franch.) Nannf. [Campanulaceae; Codonopsis radix] (*Dang shen*) 15 g *Atractylodes macrocephala* Koidz. [Asteraceae; Atractylodis macrocephalae rhizoma] (*Bai zhu*) 15 g *Cullen corylifolium* (L.) Medik. [Fabaceae; Psoraleae fructus] (*Bu gu zhi*) 15 g *Myristica fragrans* Houtt. [Myristicaceae; Myristicae semen] (*Rou dou kou*) 6 g *Euodia rutaecarpa* (Juss.) Benth. [Rutaceae; Euodiae fructus] (*Wu zhu yu*) 6 g *Cinnamomum cassia* (L.) J.Presl [Lauraceae; Cinnamomi cortex] (*Rou gui*) 10 g *Zingiber officinale* Roscoe [Zingiberaceae; Zingiberis rhizoma praeparatum] (*Pao jiang*) 10 g *Aconitum carmichaelii* Debeaux [Ranunculaceae; Aconiti radix lateralis praeparata] (*Fu zi*) 10 g *Cyperus rotundus* L. [Cyperaceae; Cyperi rhizoma] (*Xiang fu*) 12 g *Curcuma phaeocaulis* Valeton [Zingiberaceae; Curcumae rhizoma] (*E zhu*) 12 g *Salvia miltiorrhiza* Bunge [Lamiaceae; Salviae miltiorrhizae radix et rhizoma] (*Dan shen*) 15 g *Cremastra appendiculata* (D.Don) Makino [Orchidaceae; Cremastrae pseudobulbus] (*Shan ci gu*) 10 g *Actinidia chinensis* Planch. [Actinidiaceae; Actinidiae chinensis radix] (*Teng li gen*) 12 g *Pteris multifida* Poir. [Pteridaceae; Pteridis multifidae herba] (*Feng wei cao*) 10 g *Eupolyphaga sinensis* Walker [Corydidae; Eupolyphaga] (*Tu bie chong*) 10 g *Gallus gallus domesticus* Brisson [Phasianidae; Galli gigerii endothelium corneum] (*Ji nei jin*) 15 g

Abbreviations: Co. LTD.: company limited; NI: no information. There were no quality control and chemical analysis reports for the formulae.

^a^ Notes: There was no information on dosage in the trials.

^b^ Compositions with the given dosage in *Shen shao* paste were made into paste and administered to participants 20 mL/day.

The top 10 botanical drugs are *Glycyrrhiza uralensis* Fisch. ex DC. [Fabaceae; Glycyrrhizae radix et rhizoma] (*Gan cao*) (n = 14), *Atractylodes macrocephala* Koidz. [Asteraceae; Atractylodis macrocephalae rhizoma] (*Bai zhu*) (n = 14), *Codonopsis pilosula* (Franch.) Nannf. [Campanulaceae; Codonopsis radix] (*Dang shen*) (n = 13), *Astragalus mongholicus* Bunge [Fabaceae; Astragali radix] (*Huang qi*) (n = 12), *Citrus reticulata* Blanco [Rutaceae; Citri reticulatae pericarpium] (*Chen pi*) (n = 10), *Prunus mume* (Sieb.) Sieb.et Zucc. [Rosaceae; Mume fructus] (*Wu mei*) (n = 10), *Poria cocos* (Schw.) Wolf [Polyporaceae; Poria] (*Fu ling*) (n = 9), *Coix lacryma-jobi* L.var.*ma-yuen* (Roman.) Stapf [Poaceae; Coicis semen] (*Yi yi ren*) (n = 9), *Scleromitrion diffusum* (Willd.) R.J.Wang [Rubiaceae; Hedyotis diffusae herba] (*Bai hua she she cao*) (n = 6), *Curcuma phaeocaulis* Valeton [Zingiberaceae; Curcumae rhizoma] (*E zhu*) (n = 6), and *Panax notoginseng* (Burkill) F.H.Chen [Araliaceae; Notoginseng radix et rhizome] (*San qi*) (n = 5) (Supplementary Table S1). These botanical drugs were given within the recommended dosages, according to the Chinese pharmacopoeia (Commission, 2020), and are mainly divided into two types: strengthening spleen and anti-tumour capability. The top seven commonly used botanical drugs contained *Si jun zi* decoction (*Dang shen, Bai zhu, Fu ling,* and *Gan cao*)—a typical formula for gastrointestinal disorders. Six studies used these four botanical drugs*. Si jun zi* decoction originated from “Prescriptions of the People’s Welfare Pharmacy (*Tai ping hui min he ji ju fang*) (1151 AD) and is used for spleen deficiency syndrome. Modern experiments showed that *Si jun zi* decoction had anti-tumour effects on colon and lung cancer (Zhou et al., 2019; Shao et al., 2022). One ongoing multicentre, placebo-controlled trial is testing *Si jun zi* decoction granule as an intervention to prevent CRA recurrence (Ni et al., 2023). The names of botanical drugs were sourced from Kew Science and the Chinese pharmacopoeia (Commission, 2020; Trustees of the Royal Botanic Gardens, 2024). We followed the guidelines to report the botanical drugs and formulae and used the ChnPhYMO tool for assessment (Supplementary ConPhyMP checklists Supplementary Tables S1, S2) (Heinrich et al., 2022).

### 3.3 Risk of bias assessment

**FIGURE 2:**
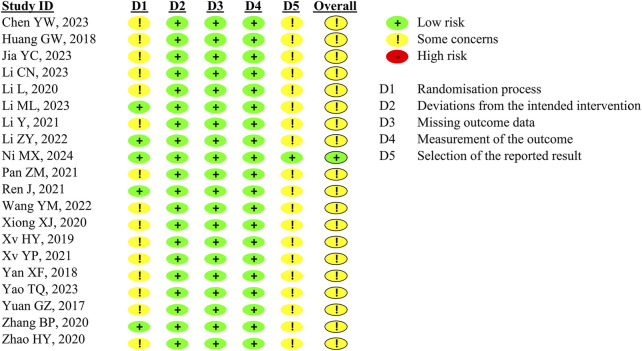
Risk of bias assessment for the primary outcome: CRA recurrence rate.

The risk of bias for the CRA recurrence rate and AEs was assessed. For the CRA recurrence rate (Figure 2), we have no information on the data analysis after the pre-specified statistical plan for 19 studies because these included studies did not provide protocols. One trial supplied the protocol (Ni et al., 2024). Therefore, we rated the selective reporting domain of 19 studies as ‘some concern.’ We also judged the randomisation process domain as some concern in 15 studies due to insufficient detail about randomisation. Three RCTs that used a placebo were assessed. Two mentioned the blinding method, while another one did not. The drop-out rate for participants was acceptable, with the highest rate being 17.1%. CRA recurrence was measured by colonoscopy and pathological results, which were unlikely to have been affected by the awareness of the received treatment; therefore, we rated the studies as low risk on the domains of bias from intervention, missing data, and outcome measurement.

Twelve studies reported on AEs (Supplementary Figure S2). We judged the measurement outcome domain to be high risk because the studies did not indicate whether outcome assessors were aware of the allocation. In addition, AE was a patient-reported outcome, which may be influenced by knowledge of the intervention. Overall, the 12 studies were considered at high risk of bias for AEs.

### 3.4 Effects of intervention

#### 3.4.1 Primary outcome: CRA recurrence rate

All the included studies (n = 20) reported on recurrence rate. Two of these studies reported at 3 months, nine studies at 6 months, 13 studies at 12 months after polypectomy, and one for cumulative recurrence rate during the 2-year follow-up.

##### 3.4.1.1 CRA recurrence rate at 12 months after polypectomy

**FIGURE 3:**
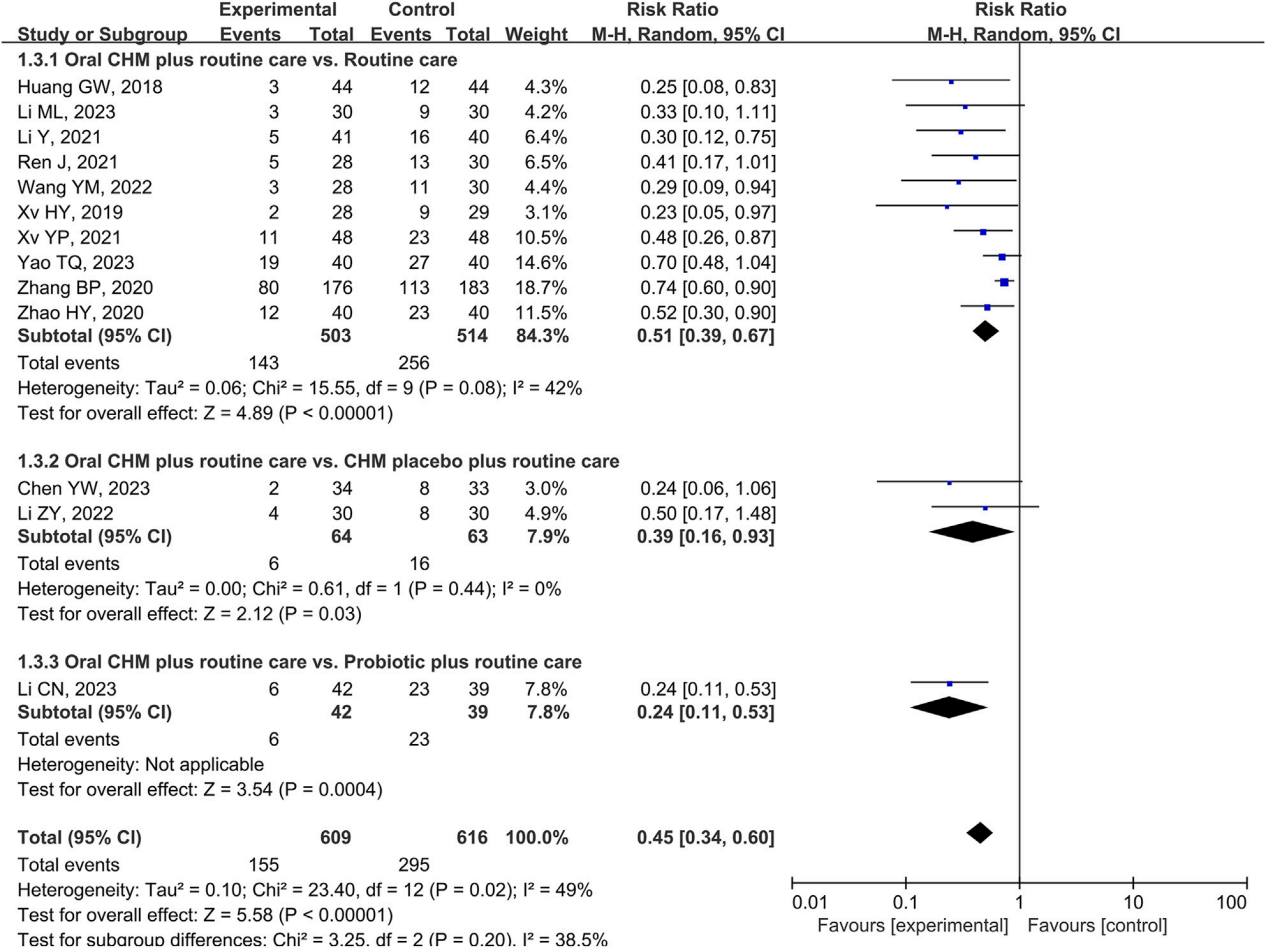
Forest plot of the CRA recurrence rate at 12 months after polypectomy.

Compared to RC alone, RC plus CHM had a 22% decreased risk of developing recurrence of CRA at 12 months after polypectomy (RR 95%CI 0.51, [0.39, 0.67], *p* < 0.00001, I^2^ = 42%, 10 studies, 1,017 participants, certainty of evidence: low) (Figure 3) (Huang et al., 2018; Xv, 2019; Zhang et al., 2020; Zhao, 2020; Li Y. et al., 2021; Ren, 2021; Xv et al., 2021; Wang, 2022; Li M. et al., 2023; Yao et al., 2023).

Compared to RC plus placebo, CHM plus RC had a 16% decreased risk of developing recurrence of CRA at 12 months after polypectomy (2 studies of *San zi* granule, 127 participants, (RR 95%CI 0.39, [0.16, 0.93], *p* < 0.03, *I* ^*2*^ = 0%, 2 studies of *San zi* granule, 127 participants, certainty of evidence: low) (Figure 3) (Li et al., 2022; Chen et al., 2023).

Compared to probiotic plus RC, CHM plus probiotic plus RC had a 45% decreased risk of developing recurrence of CRA at 12 months after polypectomy (RR 95%CI 0.24, [0.11, 0.53], 1 study, 81 participants, certainty of evidence: low) (Figure 3) (Li et al., 2023a).

Subgroup analysis was performed based on treatment duration in nine studies (Xv, 2019; Zhang et al., 2020; Zhao, 2020; Li Y. et al., 2021; Ren, 2021; Xv et al., 2021; Wang, 2022; Li M. et al., 2023; Yao et al., 2023) because one study did not mention the treatment duration (Huang et al., 2018). After 2 months of treatment or less, compared to RC alone, CHM plus RC had a 27% decreased risk of recurrence at 12 months after polypectomy (RR 0.49, [0.31, 0.78] I^2^ = 0%, 2 studies, 138 participants). After 3 months of treatment or more, there was a 21% decreased risk of recurrence (RR 0.53, [0.39, 0.74], I^2^ = 47%, 7 studies, 791 participants). There was no significant difference between these groups (Table 3; Supplementary Figure S3.1).

**TABLE 3:** Summary results of meta-analysis and additional analyses.

CRA recurrence rate	N RCTs (participants)	RR 95%CI	I^2^	RD 95%CI	I^2^	% reduction in risk
Results for CRA recurrence rate at 12 months after polypectomy						
Oral CHM plus routine care vs. Routine care	10 (1017)	0.51 [0.39, 0.67]^a^	I^2^ = 42%	−0.22 [-0.27, −0.16]^a^	I^2^ = 0%	22
Oral CHM plus routine care vs. CHM placebo plus routine care	2 (127)	0.39 [0.16, 0.93]^a^	I^2^ = 0%	−0.16 [-0.29, −0.04]^a^	I^2^ = 0%	16
Oral CHM plus routine care vs. Probiotic plus routine care	1 (81)	0.24, [0.11, 0.53]^a^	NA	−0.45 [-0.63, −0.26]^a^	NA	45
Grouped by treatment duration (oral CHM plus routine care vs. routine care)						
2 months (8 weeks) or less	2 (138)	0.49, [0.31, 0.78]^a^	I^2^ = 0%	−0.27 [-0.42, −0.11]^a^	I^2^ = 0%	27
3 months (12 weeks) or longer	7 (791)	0.53, [0.39, 0.74]^a^	I^2^ = 47%	−0.21 [-0.27, −0.15]^a^	I^2^ = 0%	21
Grouped by use of CM syndrome or no mention of CM syndrome (oral CHM plus routine care vs. routine care)						
Indicate CM syndrome differentiation	6 (459)	0.52 [0.39, 0.70]^a^	I^2^ = 13%	−0.23 [-0.31, −0.16]^a^	I^2^ = 0%	23
Not indicate CM syndrome differentiation	5 (558)	0.45 [0.24, 0.84]^a^	I^2^ = 60%	−0.20 [-0.28, −0.13]^a^	I^2^ = 0%	20
Specific CHM formula (oral CHM plus routine care vs. routine care)						
*Tiao chang xiao liu* formula	2 (440)	0.53 [0.23, 1.24]	I^2^ = 72%	−0.20 [-0.30, −0.09]^a^	I^2^ = 16%	20
Includes *Si jun zi* decoction	3 (203)	0.26 [0.13, 0.54]^a^	I^2^ = 0%	−0.23 [-0.33, −0.12]^a^	I^2^ = 0%	23
*San zi* granule	2 (127)	0.39, [0.16, 0.93]^a^	I^2^ = 0%	−0.16 [-0.29, −0.04]^a^	I^2^ = 0%	16
Results for CRA recurrence rate at 6 months after polypectomy						
Oral CHM plus routine care vs. Routine care	8 (764)	0.44 [0.36, 0.55]^a^	I^2^ = 0%	−0.23 [-0.34, −0.12]^a^	I^2^ = 71%	23
Oral CHM plus routine care vs. CHM placebo plus routine care	1 (60)	0.50 [0.10, 2.53]	NA	−0.07 [-0.22, 0.08]	NA	7
Grouped by treatment duration (oral CHM plus routine care vs. routine care)						
2 months (8 weeks) or less	3 (375)	0.41 [0.31, 0.55]^a^	I^2^ = 0%	−0.28 [-0.52, −0.04]^a^	I^2^ = 89%	28
3 months (12 weeks) or longer	5 (389)	0.48 [0.35, 0.67]^a^	I^2^ = 0%	−0.18 [-0.26, −0.10]^a^	I^2^ = 1%	18
Grouped by use of CM syndrome or no mention of CM syndrome (oral CHM plus routine care vs. routine care)						
Indicate CM syndrome differentiation	5 (417)	0.50 [0.37, 0.68]^a^	I^2^ = 0%	−0.19 [-0.26, −0.11]^a^	I^2^ = 0%	19
Not indicate CM syndrome differentiation	3 (300)	0.42 [0.26, 0.66]^a^	I^2^ = 0%	−0.17 [-0.27, −0.06]^a^	I^2^ = 27%	17
Specific CHM formula (oral CHM plus routine care vs. routine care)						
Includes *Si jun zi* decoction	3 (261)	0.49 [0.32, 0.74]^a^	I^2^ = 0%	−0.17 [-0.27, −0.08]^a^	I^2^ = 0%	17
*San zi* granule	1 (60)	0.50 [0.10, 2.53]	NA	−0.07 [-0.22, 0.08]	NA	7
Results for the CRA recurrence rate at 3 months after polypectomy						
Oral CHM plus routine care vs. Routine care	2 (202)	0.46 [0.22, 0.96]^a^	I^2^ = 0%	−0.09 [-0.27, 0.09]	I^2^ = 75%	9
Results for the CRA recurrence rate during the 2-year follow-up after polypectomy						
Oral CHM plus routine care vs. CHM placebo plus routine care	1 (336)	0.73 [0.58, 0.90]^a^	N/A	−0.16 [-0.27, −0.06]^a^	N/A	16
Results for adverse events during the follow-up after polypectomy						
Oral CHM plus routine care vs. routine care	10 (1079)	0.78 [0.30, 1.99]	I^2^ = 0%	−0.00 [-0.01, 0.01]	I^2^ = 0%	0
Oral CHM plus routine care vs. CHM placebo plus routine care	2 (467)	1.13 [0.44, 2.86]	N/A	0.00 [-0.03, 0.04]	I^2^ = 0%	0

Abbreviations: CHM, Chinese herbal medicine; CI, confidence interval; CM, Chinese medicine; CRA, colorectal adenoma; RR, risk ratio; RD, risk difference.

^a^ Note: Statistically significant difference between groups.

For the use of CM syndrome differentiation or no mention of syndrome differentiation, there was a decreased risk of recurrence at 12 months for CHM plus RC in each group with no significant difference between these groups (Table 3; Supplementary Figure S3.2).

For *Tiao chang xiao liu* formula, there was no significant difference between *Tiao chang xiao liu* formula plus RC and RC alone (RR 0.53, [0.23, 1.24], *p* = 0.06, I^2^ = 72%, 2 studies, 440 participants), but the heterogeneity was considerable (Supplementary Figure S3.3) (Zhang et al., 2020; Li Y. et al., 2021).

According to the commonly used botanical drugs in the interventions, we performed analysis based on including *Si jun zi* decoction, whose ingredients were listed in the top 10 used botanical drugs. Compared to RC alone, CHMs that included *Si jun zi* decoction plus RC had a 23% lower CRA recurrence rate (RR 0.26, [0.13, 0.54] I^2^ = 0%, 3 studies, 203 participants) (Supplementary Figure S3.5).

Sensitivity analyses showed that after omitting any one study of CRA recurrence rate at 12 months after polypectomy, the results were similar to the overall pooled analysis, indicating no strong effect on the overall result from any single study was observed (Table 3; Supplementary Figure S4.1; Supplementary Supplementary Table S4.1).

##### 3.4.1.2 CRA recurrence rate at other time points (at 6 and 3 months after polypectomy, and during the 2-year follow-up after polypectomy)

**FIGURE 4:**
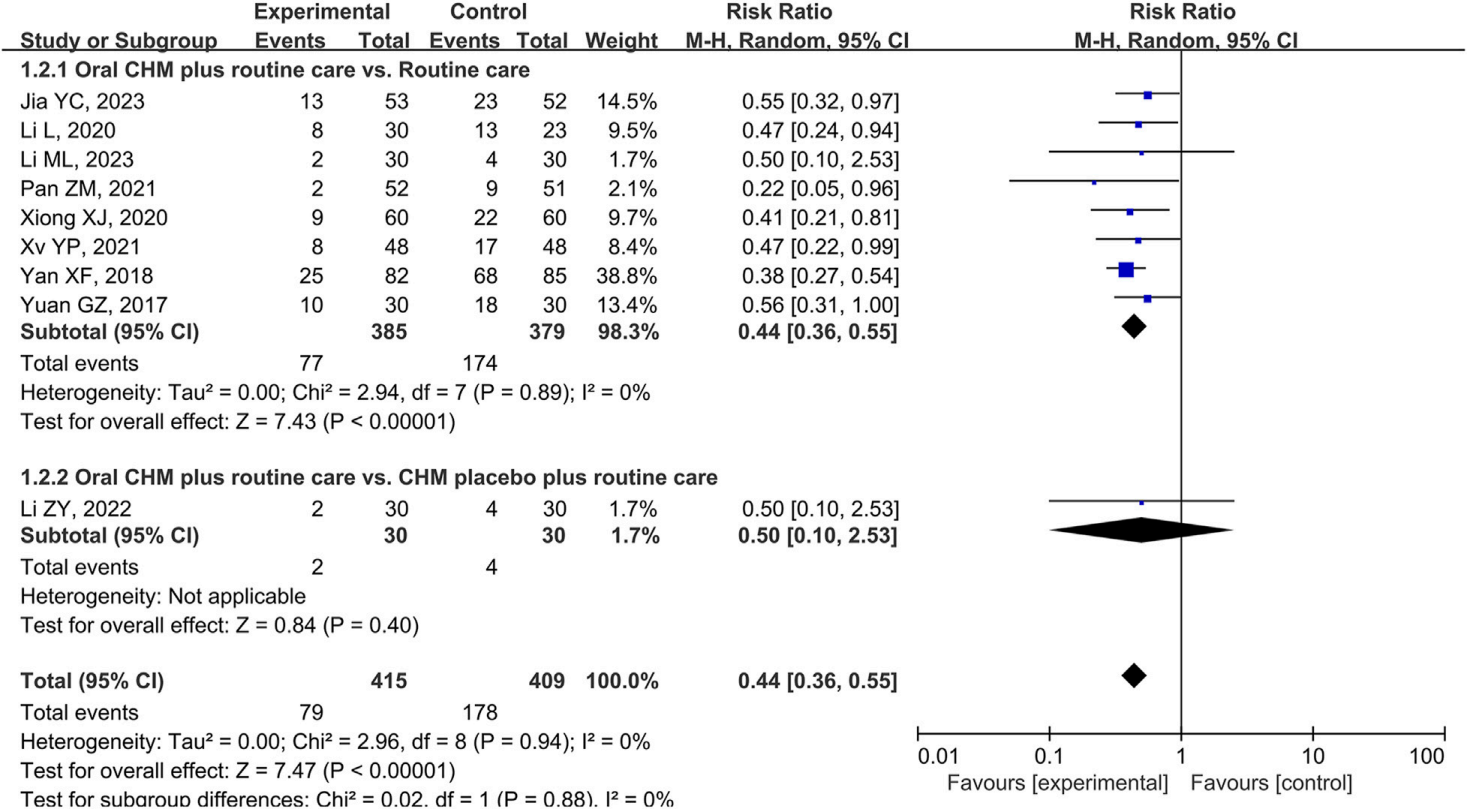
Forest plot of the CRA recurrence rate at 6 months after polypectomy.

At 6 months after polypectomy, compared to RC alone, CHM plus RC had a 23% reduction in CRA recurrence (RR 0.44, [0.36, 0.55], *p* < 0.00001, I^2^ = 0%, 8 studies, 764 participants, certainty of evidence: moderate) (Figure 4) (Yuan, 2017; Yan et al., 2018; Li et al., 2020a; Xiong and Gu, 2020; Pan, 2021; Xv et al., 2021; Jia et al., 2023; Li et al., 2023b).

There was no significant difference between groups that received oral CHM plus RC or CHM placebo plus RC at 6 months after polypectomy (RR 0.50, [0.10, 2.53], *p* = 0.40, 1 study, 60 participants, certainty of evidence: low) (Figure 4) (Li et al., 2022).

 The results of subgroup analyses for treatment duration and use of CM syndrome differentiation favoured the oral CHM plus RC groups compared with RC alone with no significant differences between the subgroups. CHMs that included Si jun zi decoction plus RC showed significant reductions in risk compared to RC alone at 6 months following polypectomy, but this group was not significantly different to the formulae that did not include Si jun zi decoction. (Table 3; Supplementary Figures S3.6, S3.7, S3.8).

Sensitivity analyses showed a slight difference in CRA recurrence at 6 months after polypectomy between the combined RR after omitting any given study and the total combined RR with all included studies, suggesting the pooled result was stable (Table 3; Supplementary Figure S4.2; Supplementary Table S4.2). At 3 months after polypectomy, compared to RC alone, CHM plus RC had a 9% reduction in recurrence rate (RR 0.46, [0.22, 0.96], *p* = 0.04, I^2^ = 0%, 2 studies, 201 participants, certainty of evidence: low) (Supplementary Figure S5) (Xv et al., 2021; Jia et al., 2023).

One trial of *Shen bai* granule reported the adenoma recurrence rate by calculating the proportion of participants with at least one recurrent adenoma at any repeated colonoscopies (two in total) during a 2-year follow-up (Ni et al., 2024). Compared to RC plus CHM placebo, CHM plus RC had a 16% reduction in the recurrence rate (RR 0.73, [0.58, 0.90], *p* = 0.004, 1 study, 336 participants, certainty of evidence: moderate) (Supplementary Figure S6).

#### 3.4.2 Secondary outcomes

Numbers of people who had one or two CRAs, or three and more than three CRAs, were not reported by the included studies. Two studies reported the number of advanced CRAs, showing that there was no significant difference between the integrative medicine groups and the control groups (Zhang et al., 2020; Ni et al., 2024). One RCT, comparing oral CHM plus RC with RC showed no significant difference in recurrence of CRA with 1 cm size or larger between the two groups (Yao et al., 2023). Two studies reported on the diagnosis of CRC. None of the participants were newly diagnosed with cancer (Xv et al., 2021; Chen et al., 2023).

##### 3.4.2.1 Safety of oral CHM for CRA recurrence

Twelve studies (n = 1,546) assessed AEs as a safety outcome during the study period (3 months to 2 years) (Yuan, 2017; Xv, 2019; Li L. et al., 2020; Zhang et al., 2020; Zhao, 2020; Li Y. et al., 2021; Ren, 2021; Xv et al., 2021; Wang, 2022; Chen et al., 2023; Jia et al., 2023; Ni et al., 2024). Among them, nine studies showed no AEs occurred in treatment and control groups : during the trials (3‐12 months) (Yuan, 2017; Xv, 2019; Li L. et al., 2020; Zhang et al., 2020; Zhao, 2020; Li Y. et al., 2021; Ren, 2021; Wang, 2022; Chen et al., 2023), with one study comparing oral CHM plus RC to CHM placebo plus RC (Chen et al., 2023) and eight studies comparing oral CHM plus RC to RC (Yuan, 2017; Xv, 2019; Li L. et al., 2020; Zhang et al., 2020; Zhao, 2020; Li Y. et al., 2021; Ren, 2021; Wang, 2022). Two studies comparing oral CHM plus RC to RC reported 16 minor and transient AEs (Xv et al., 2021; Jia et al., 2023), with seven in the oral CHM plus RC group and nine in the RC group. This was not a significant difference (RR 0.78, [0.30, 1.99], *p* = 0.60, I^2^ = 0%) (Supplementary Table S11.1). One trial (oral CHM plus RC versus CHM placebo plus RC) reported 17 AEs (during 2 years) with no significant difference between groups (RR 1.13, [0.44, 2.86], *p* = 0.80) (Ni et al., 2024) (Supplementary Table S11.2). These AEs included vomiting, skin rash, nausea, abdominal distension, diarrhoea, fatigue, and dizziness (Supplementary Figure S7).

#### 3.4.3 Publication bias

The funnel plot of CRA recurrence rate in comparing oral CHM plus RC and RC at 12 months after polypectomy showed an obvious asymmetry, suggesting potential publication bias [(Supplementary Figure S8) and Egger’s test (t 11.34 [2.30, -1.52], (*p* < 0.000)] (Supplementary Figure S9.1; Supplementary Table S9.1). As a result of less than 10 included trials, other outcome measures were not assessed for publication bias.

## 4 Discussion

### 4.1 Summary of evidence

Our systematic review presents an up-to-date and comprehensive review of oral CHM treatment for CRA recurrence after polypectomy. We identified 20 RCTs through nine English and Chinese databases and conducted meta-analysis to explore the effects of oral CHMs for CRA recurrence. Our findings suggest that compared to RC alone, oral CHM plus RC was associated with a lower CRA recurrence rate at 3 months, 6 months, and 12 months after polypectomy with low to moderate levels of certainty. Compared to CHM placebo plus RC, oral San zi granule plus RC was associated with lower risk of CRA recurrence at 12 months and at 2 years follow up. For AEs, there was no significant difference between oral CHM plus RC versus RC, and oral CHM plus RC versus CHM placebo plus RC, suggesting that oral CHMs were well tolerated in people following surgery for CRA.

CRA management is an integral part of cancer prevention. CHM shows potential effects on CRC (Chen et al., 2016) and adenoma management (Zhang et al., 2021). In addition, a meta-analysis reported that combining traditional oriental herbal medicine and conventional medications improved the level of natural killer cells compared to conventional medications, suggesting that the herbal medicines enhanced the immune function of cancer patients (Bae et al., 2017). Similar systematic reviews comparing oral CHM plus RC with RC revealed that oral CHM lowered the risk for colorectal polyps’ recurrence (Chen S. et al., 2020; Lin et al., 2020; Zhou et al., 2020).

Our findings further add to the published literature. The primary outcome (CRA recurrence rate) is defined as the number of participants with recurrent adenoma divided by the number of participants with adenoma at baseline colonoscopy, according to the current Chinese consensus (Fang et al., 2021). Previous meta-analyses assessing oral CHM for CRA recurrence did not use this precise definition of CRA recurrence rate (Lin et al., 2020). Therefore, our study is the first review to use this definition of recurrence rate to analyse the current evidence on oral CHM for CRA recurrence prevention.

### 4.2 Effects of oral CHM and their potential mechanisms

In our study, only 3 out of 19 included studies used a CHM placebo in the control group (Li et al., 2022; Chen et al., 2023; Ni et al., 2024), suggesting that more placebo-controlled trials can be conducted to explore the efficacy of oral CHM for CRA recurrence. Placebo control may provide better scientific quality and reliability of results (Krol et al., 2020). The two placebo-controlled studies that used *San zi* granule combined with RC showed a significantly greater reduction in risk of CRA recurrence at 12 months in the pooled result (Figure 4). *San zi* granule is composed of nine botanical drugs, such as *Wu mei*, *Huang qi*, and other botanical drugs. A recent experiment reported that *San zi* granule altered the gut microbiome, including an increase in probiotics and a decrease in pernicious bacteria, and changed the levels of metabolites related to primary bile acid biosynthesis and taurine metabolism (Shang et al., 2022). The gut microbiome and relevant metabolites lead to gene expression disorders, changes in intestinal barrier, and activation of inflammation and lipopolysaccharide metabolism, which play essential roles in carcinogenesis (Ağagündüz et al., 2023). Furthermore, research into roles of micro-RNAs in regulating genes associated with cancer suppression has shown that bioactive substances in foods and particular dietary models may regulate the immune system, reduce pro-inflammatory signalling, and modulate the gut microbiome (Şahin et al., 2023). Future studies of CHMs could investigate effects on the microbiome and micro-RNAs.

We analysed subgroups to explore the effects of oral CHM plus RC in included studies with similar characteristics. For treatment duration, our meta-analysis showed that oral CHM plus RC may reduce the risk of CRA recurrence, irrespective of shorter (two months or less) or longer (three months or more) treatment duration. Expert consensus suggested that three to 6 months of CM treatment is recommended to reduce CRA recurrence (Zhang et al., 2021). Based on our results, more evidence is needed to identify the best treatment duration to reduce CRA recurrence. Similar results were observed for subgroup analysis based on the use of CM syndrome differentiation or no mention of its use. CM theory emphasises individual treatment based on different CM syndromes (Peng et al., 2022). A previous meta-analysis showed that strengthening the spleen method prevented the recurrence of colorectal polyps (including adenomas) (Zhou et al., 2020). However, in this review, CM syndrome differentiation did not affect the risk of CRA recurrence at 6 and 12 months after polypectomy. This result may be attributed to the limited number of included studies and the predominance of spleen deficiency syndrome among people with CRA, leading to less pronounced effects of syndrome differentiation (Liu and Chen, 2010). In addition, further studies may be needed to identify the full range of CM syndromes in people with CRA in different regions in China. For the analysis based on the specific formula, we found no difference between *Tiao chang xiao liu* formula plus RC and RC alone with a high heterogeneity (I^2^ = 72%), even though each of the included studies showed a significant difference. This result was due to differing effect sizes between the small and large studies. The result favoured the intervention when we changed to the fixed-effect model (Supplementary Figure S3.4). So, it calls for more clinical studies on the *Tiao chang xiao liu* formula.

**FIGURE 5:**
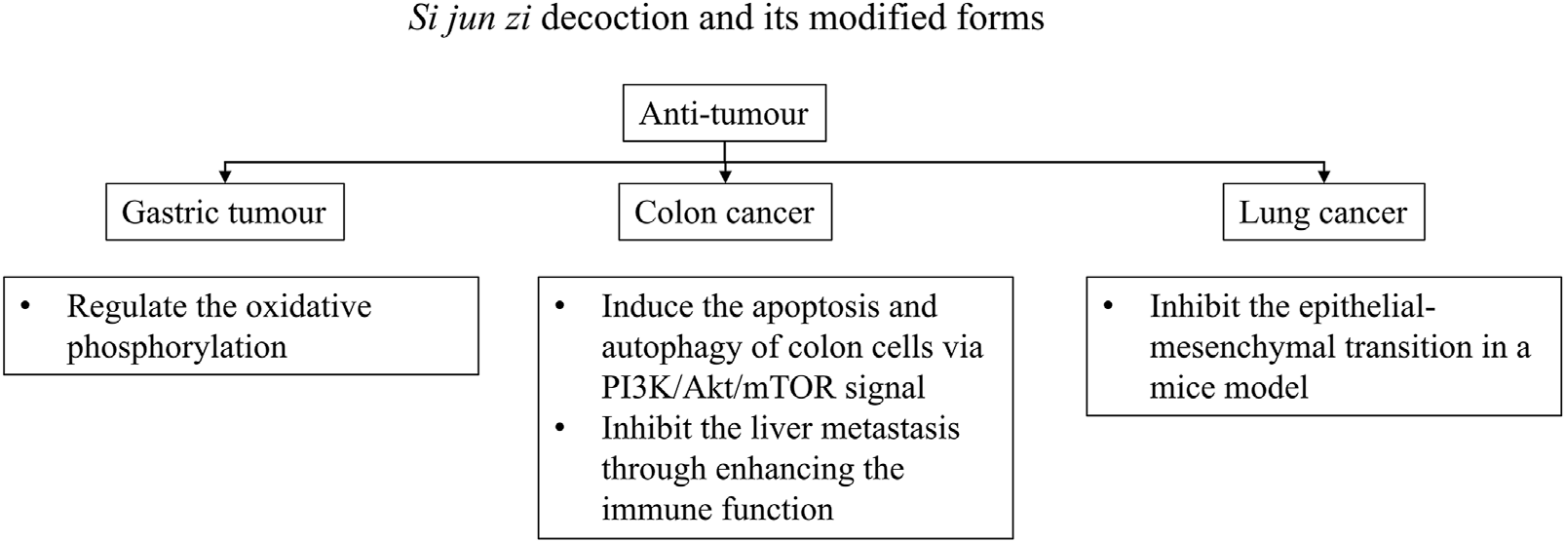
Potential anti-tumour mechanisms of *Si jun zi* decoction and its modified forms.

We calculated the frequency of botanical drugs in the intervention and found that botanical drugs to strengthen the spleen and *qi* and suppress tumours were commonly used for CRA recurrence. The top six botanical drugs included the ingredients of *Si jun zi* decoction, which is a typical formula to strengthen the spleen and *qi*. Its effects have been associated with intestinal mucosal restoration (Shi et al., 2019). In its original version, *Si jun zi* decoction contained *Panax ginseng* C.A.Mey. [Araliaceae; Ginseng radix et rhizoma] (*Ren shen*), *Fu ling*, *Bai zhu*, and *Gan cao*, but in clinical practice and research, *Dang shen* is usually selected as a substitute for *Ren shen*. Previous research demonstrated that *Si jun zi* decoction or its modified form accelerated the apoptosis and autophagy of colon cells (Shang et al., 2023) and blocked the liver metastasis of colon cancer (Zhou et al., 2019). As for other anti-tumour activities, the original or modified *Si jun zi* decoction influenced oxidative phosphorylation to reduce gastric tumour growth (Zhu et al., 2024) and prevented epithelial–mesenchymal transition in a mouse model of non-small cell lung cancer (Shao et al., 2022) (Figure 5). Modification of *Si jun zi* decoction includes addition of *Atractylodes lancea* (Thunb.) DC. [Asteraceae; Atractylodis rhizoma] (*Cang zhu*), *Citrus reticulata* Blanco [Rutaceae; Citri reticulatae pericarpium] (*Chen pi*), and *Citrus × aurantium* L. [Rutaceae; Aurantii fructus] (*Zhi ke*) based on the individualized treatment. A major compound in *Cang zhu* is atractylodin, which was identified as an inhibitor of N-acylethanolamine-hydrolysing acid amidase (NAAA) that can upregulate palmitoylethanolamide (PEA), reduce microglial activation, and suppress inflammatory responses (Yang et al., 2020). A recent study showed that PEA, a fatty acid amide, restricted the proliferation, differentiation, and migration of colorectal tumour cells and induced cell cycle arrest in the G2/M phase (Pagano et al., 2021). NAAA, the main PEA hydrolytic enzyme, plays an important role in human colon cancer. In mice, an NAAA inhibitor reduced tumour growth and Ki-67 expression, a marker of cell proliferation (Romano et al., 2022). *Chen pi*, which is in the list of commonly used herbs, and *Zhi ke* are sources of bergamottin (Gao et al., 2022), a type of furanocoumarin (Ahmed et al., 2020). Furanocoumarins from natural products are reported to exert anti-tumour effects via apoptosis of malignant tumour cells, autophagy, and cell cycle arrest (Ahmed et al., 2020). Downregulation of the nuclear factor kappa-light-chain-enhancer of activated B cells (NF-κB) pathway, the phosphatidylinositol 3-kinase/RAC-α serine/threonine-protein Kinase (PI3K/Akt) pathway and G2/M phase cell cycle arrest were potential mechanisms of bergamottin to inhibit carcinogenesis (Ahmed et al., 2020). Overall, formulae including *Si jun zi* decoction reduced CRA recurrence with low heterogeneity in our review. More research about *Si jun zi* decoction and its variants for CRA recurrence could be conducted.

**FIGURE 6:**
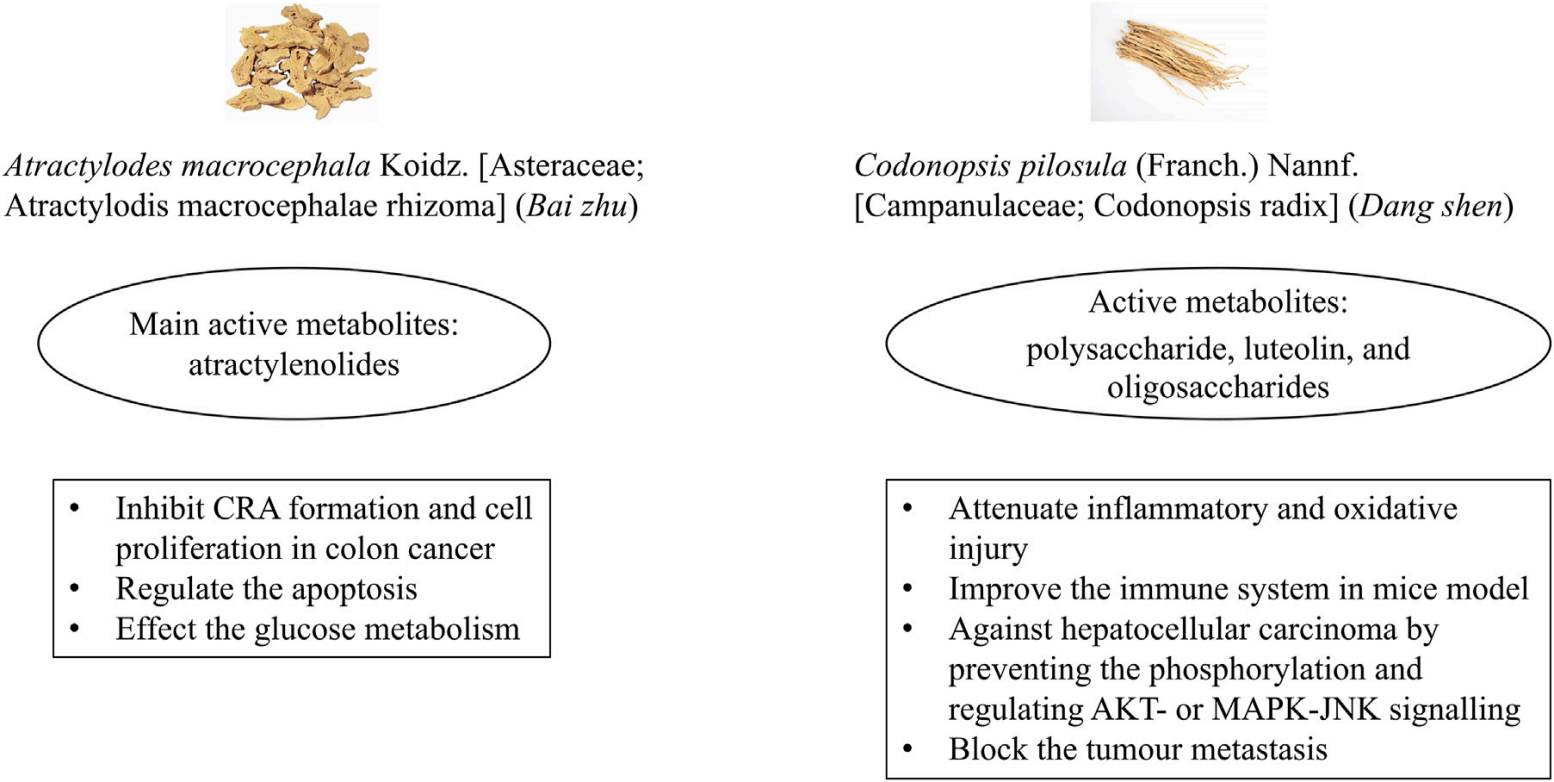
Potential mechanisms of *Bai zhu* and *Dang shen* to contribute to colorectal adenoma recurrence. Note: CRA, colorectal adenoma.

*Bai zhu* and *Dang shen* are mainly used to strengthen the spleen and *qi* and remove dampness in CM theory. In modern research, they have been shown to affect intestinal health. *Bai zhu* has been reported to improve intestinal flora and immune function, reduce inflammation, and promote apoptotic tumour cell death (Zhu et al., 2018). Active metabolites of *Bai zhu,* the atractylenolides, were reported to inhibit CRA formation (Li et al., 2018) and cell proliferation in colon cancer (Li Y. et al., 2020), as well as regulate apoptosis of colon adenocarcinoma cells via mitochondria-related signalling (Chan et al., 2020). Recent studies found that metabolites derived from *Dang shen* attenuated inflammation and oxidative injury (Zou et al., 2023), enhanced the immune system in mice (Bai et al., 2020), and inhibited the proliferation and metastasis of tumour cells (Xin et al., 2012; Yu et al., 2023). A review of research showed that *Bai zhu* contained the flavonoids apigenin and luteolin (Zhu et al., 2018). Luteolin was obtained from *Dang shen* as well (Yu et al., 2023). Apigenin had inhibitory effects on p38 phosphorylation and expression of NF-κB, blocking epithelial to mesenchymal transition in the HCT116 CRC cell line (Fernández et al., 2021). Luteolin activated antioxidant enzymes, increased Bax expression, and induced apoptosis via caspase-9 and caspase-3 in the H29 human CRC cell line (Fernández et al., 2021). Overall, *Bai zhu* and *Dang shen* are possible botanical drugs to prevent CRA recurrence due to their anti-tumour effects *in vivo* and *in vitro* (Figure 6). Few studies related to *Bai zhu* or *Dang shen* for adenoma recurrence have been conducted; further research on these issues is necessary.

In addition, some anti-tumour botanical drugs are listed in the commonly used ingredients, including *Yi yi ren*, *E zhu*, *Bai hua she she cao*, and *San qi*. Each has received research attention. In a model of pre-CRC in rats, feeding with extracts of Yi yi ren inhibited formation of colonic preneoplastic lesions (Li et al., 2011). Coix seed oil has been shown to promote apoptosis of HT-29 colon cells (Ni et al., 2021). Modern pharmacological research revealed that the combined use of *Huang qi* and *E zhu* prevented the growth of CRC cells (Bian et al., 2022). *Bai hua she she cao* also has anti-tumour effects through regulating multi-pathways, including activation of apoptosis (Zhoufan et al., 2018), downregulation of Wnt signalling (Reyes-Farias and Carrasco-Pozo, 2019), inhibition of tumour cell invasion and migration (Wu et al., 2017), anti-inflammatory effects (He et al., 2018), and enhancement of immune response (Son et al., 2017). Overexpression of the Wnt/β-catenin pathway contributed to colorectal cancer development, with a positive correlation with the level of the transient receptor potential (TRP) cation channel, subfamily melastatin (M), member 8 (TRPM8) in primary colon tumours. Inhibition of TRPM8 led to downmodulation of Wnt pathway transcription and increased expression of oncogenes (C-Myc and Cylin D1) and β-catenin, suggesting a potential treatment target in CRC (Pagano et al., 2023).

We also found some lower-frequency botanical drugs with anti-cancer effects, such as *Scutellaria barbata* D.Don [Lamiaceae; Scutellariae barbatae herba] (*Ban zhi lian*)*, Lobelia chinensis* Lour. [Campanulaceae; Lobeliae chinensis herba] (*Ban bian lian*), and *Sparganium stoloniferum* (Buch.-Ham. ex Graebn.) Buch.-Ham. ex Juz. [Typhaceae; Sparganii rhizoma] (*San leng*) (Suh et al., 2007; Jia et al., 2021; Luo et al., 2024). The intervention characteristics, many of which contained botanical drugs to strengthen the spleen plus ones with anti-tumour actions, may explain how the CHMs work to prevent CRA recurrence, even though the anti-tumour botanical drugs are not the same in each formula.

Comparing the recurrence rate (49.81%) of the control group (RC alone) at 12 months to epidemiological data in China, which showed 59.46% at follow-ups of less than and including 1 year (Gao et al., 2010), we found the results were similar. Therefore, the data in our study may reflect the real-world situation to some degree. In our review, oral CHM plus RC reduced the risk of recurrence by 22% (RD 95% CI -0.22 [-0.27, −0.16]) compared to RC alone, showing an acceptable effect of oral CHM.

We analysed sensitivity using the leave-one-out method and examined studies with different characteristics. The effects of omitting any one study were similar to the total effect of all the included studies for CRA recurrence at 12 months post-polypectomy. This suggests our results were not greatly influenced by any single study.

Specific CRA characteristics increase the risk of developing cancer, including adenoma size equal to or exceeding 10 mm, pathological examination showing villous or tubulovillous, high-grade dysplasia, and three or more adenomas (Gupta et al., 2020). However, few included studies assessed CRA characteristics in the follow-up colonoscopy (Zhang et al., 2020; Yao et al., 2023). Future trials should consider the assessment of CRA characteristics as an outcome when exploring CHM effects.

Potential publication bias was observed in studies comparing oral CHM plus RC to RC on CRA recurrence at 12 months after polypectomy. This may be due to the limited number of included studies or the higher chance of publication of studies with positive results.

### 4.3 Limitations

This paper has limitations. First, we did not search RCT registries or ongoing studies, although we included other types of studies (published articles, theses, and conference papers) to collect a broad range of evidence. Second, the included studies presented “some concerns” on the risk of bias for CRA recurrence, mainly due to insufficient details for randomisation and allocation concealment, protocol registration, and protocol publication. In addition, it is challenging to implement the blinding process when using RC as a control. Participants may know their assigned group if they received oral CHM decoction. Consequently, judgement of risk of bias led to the downgrade of certainty of evidence in our meta-analysis. Finally, we could not assess the pooled result for the effectiveness of oral CHM on advanced CRA because few studies reported this outcome. CRA progression is an essential clinical feature for CRC development and could be investigated in future clinical trials. The growth of colorectal tumours is related to a series of complex pathways. Although consumption of CHMs may modulate some mechanisms in CRC development and become potential botanical drugs, dietary factors and the condition of the microbiome also play important roles.

## 5 Conclusion

Our systematic review and meta-analysis suggest there is low-to-moderate-level evidence that oral CHM plus RC is beneficial in reducing CRA recurrence and is well-tolerated in people with CRA. Based on previous pharmacological research, our results suggest that *San zi* granule and *Si jun zi* decoction potentially serve as the representative formulae for CRA recurrence prevention. Experimental studies on the frequent botanical drugs have found anti-cancer effects that may explain their mechanisms. However, due to the limited number of clinical studies and insufficient evidence level, it is recommended that large-scale, rigorously designed RCTs could be conducted in the future to provide higher-level clinical evidence.

## Data Availability

The original contributions presented in the study are included in the article/supplementary material, further inquiries can be directed to the corresponding authors.

## Abbreviations

AE, adverse event; AMED, The Allied and Complementary Medicine Database; CHM, Chinese herbal medicine; CI, confidence interval; CRA, colorectal adenoma; CINAHL, Cumulative Index to Nursing and Allied Health Literature; CM, Chinese medicine; GRADE, Grading of Recommendations, Assessment, Development and Evaluations; MeSH, medical subject headings; PRISMA, Preferred Reporting Items for Systematic Reviews and Meta-Analyses; RC, routine care; RCT, randomised controlled trial; RoB 2, risk of bias 2; RR, risk ratio.
