# Supplementary material for: Effects of Chinese herbal medicine on colorectal adenoma recurrence following polypectomy: a systematic review and meta-analysis
Source: Front Pharmacol. 2025 Mar 20;16:1460900. doi: 10.3389/fphar.2025.1460900 (PMC11966114; doi:10.3389/fphar.2025.1460900)
Supplement: Supplementary file 3 [file DataSheet1.docx]

Supplemental material

1 The most commonly used botanical drugs in CHMs for CRA recurrence

2 Risk of bias assessment for adverse events (AEs)

3 Additional analyses

4 Sensitivity analyses

5 Forest plot CRA recurrence rate at three months after polypectomy

6 Forest plot of CRA recurrence rate during 2-year follow-up after polypectomy

7 Forest plot of AEs during the follow-up period

8 Funnel plots

9 Egger’s test

10 Search strategy

11 Information of AEs

12 GRADE assessments

# 1 The most commonly used botanical drugs in CHMs for CRA recurrence

Table S1 The most commonly used botanical drugs in CHMs for CRA recurrence

| Frequency | *Pin yin* name^*^ | Botanical drug name^**^ |
| --- | --- | --- |
| 14 | *Gan cao* | *Glycyrrhiza uralensis* Fisch. ex DC. [Fabaceae; Glycyrrhizae radix et rhizoma] |
| 14 | *Bai zhu* | *Atractylodes macrocephala* Koidz. [Asteraceae; Atractylodis macrocephalae rhizoma] |
| 13 | *Dang shen* | *Codonopsis pilosula* (Franch.) Nannf. [Campanulaceae; Codonopsis radix] |
| 12 | *Huang qi* | *Astragalus mongholicus* Bunge [Fabaceae; Astragali radix] |
| 10 | *Chen pi* | *Citrus reticulata* Blanco [Rutaceae; Citri reticulatae pericarpium] |
| 10 | *Wu mei* | *Prunus mume* (Sieb.) Sieb.et Zucc. [Rosaceae; Mume fructus] |
| 9 | *Fu ling* | *Poria cocos* (Schw.) Wolf [Polyporaceae; Poria] |
| 9 | *Yi yi ren* | *Coix lacryma-jobi* L.var.*ma-yuen* (Roman.) Stapf [Poaceae; Coicis semen] |
| 6 | *Bai hua she she cao* | *Scleromitrion diffusum* (Willd.) R.J.Wang [Rubiaceae; Hedyotis diffusae herba] (aka *Hedyotis diffusa* Willd.) |
| 6 | *E zhu* | *Curcuma phaeocaulis*Valeton [Zingiberaceae; Curcumae rhizoma] |
| 5 | *San qi* | *Panax notoginseng* (Burkill) F.H.Chen [Araliaceae; Notoginseng radix et rhizome] |

Note: abbreviations: aka, also known as; CHM, Chinese herbal medicine; CRA, colorectal adenoma.

^*^*Pin yin* names were standardized based on the 2020 Pharmacopoeia of the People’s Republic of China (https://db.ouryao.com/yd2020/ accessed 3 July 2024).

^**^Botanical drug name names are sourced from the “Kew Science” ([Home page - Medicinal Plant Names Services (kew.org)](https://mpns.science.kew.org/mpns-portal/) accessed 3 July 2024)

# 2 Risk of bias assessment for adverse events (AEs)

Figure S2 Risk of bias assessment for AEs


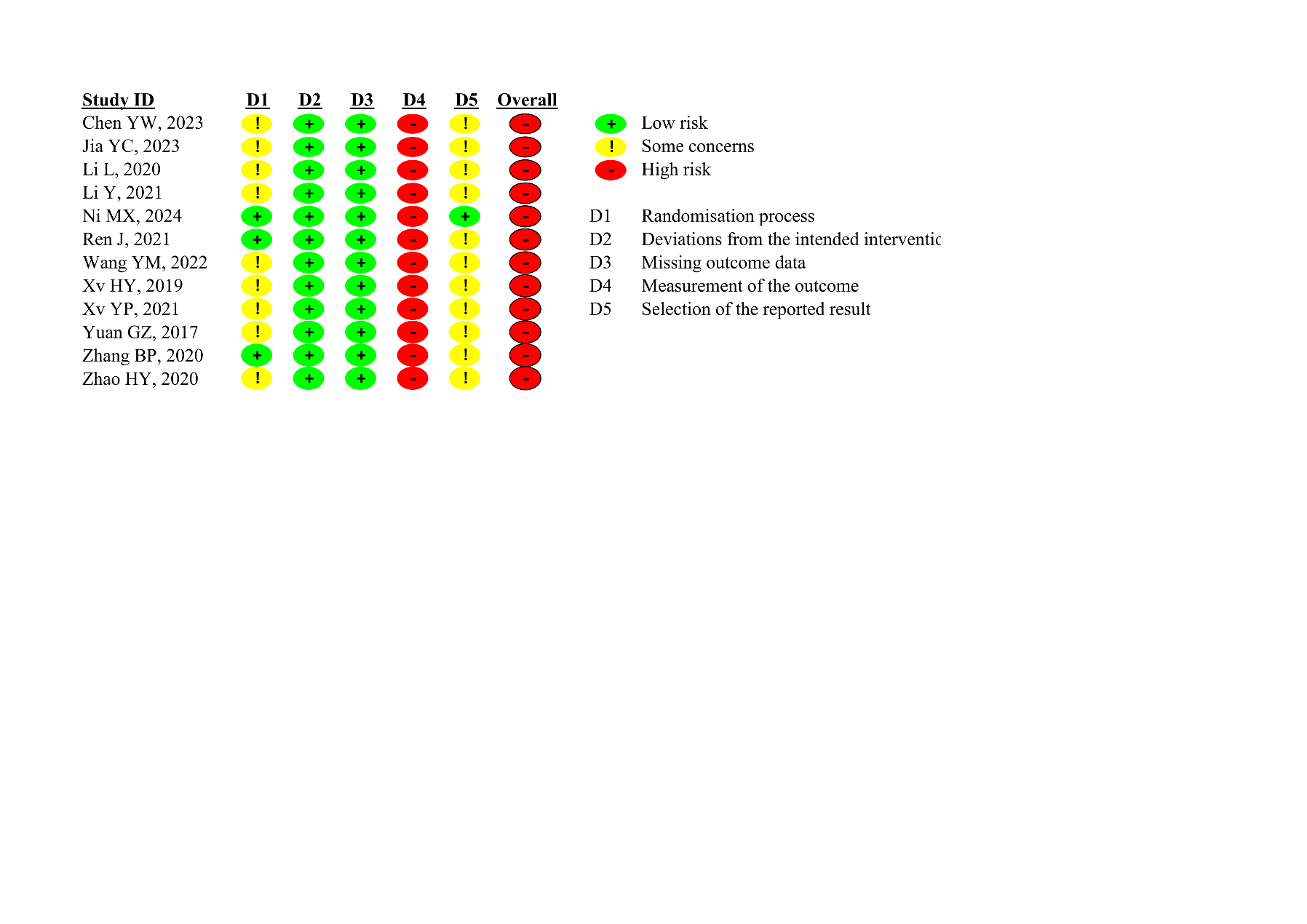


# 3 Additional analyses

Figure S3.1 Forest plot of colorectal adenoma (CRA) recurrence rate at 12 months for oral Chinese herbal medicine (CHM) plus routine care (RC) versus RC with groups for treatment duration


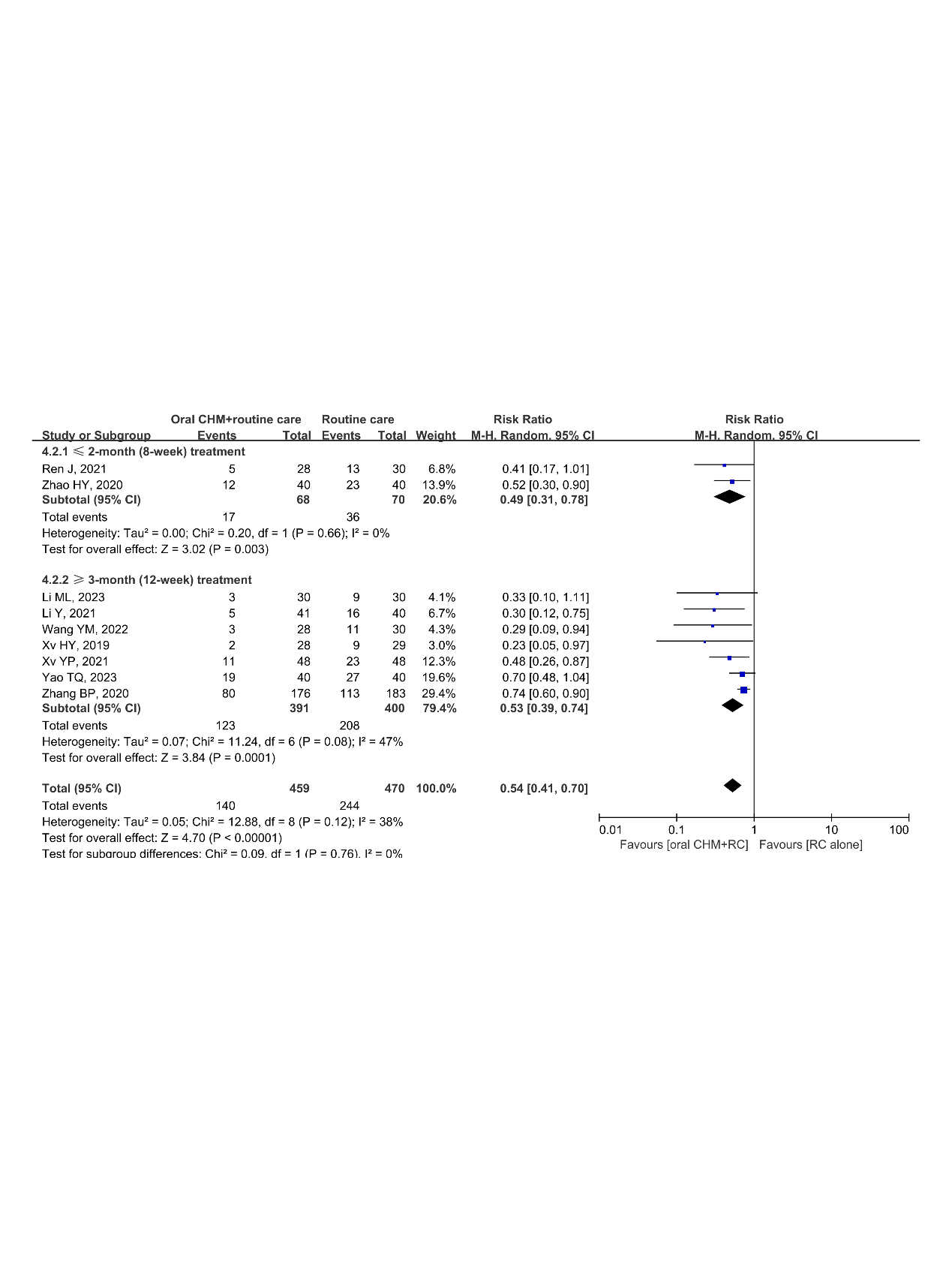


Figure S3.2 Forest plot of CRA recurrence rate at 12 months for oral CHM plus RC versus RC alone with groups for use of Chinese medicine (CM) syndrome differentiation


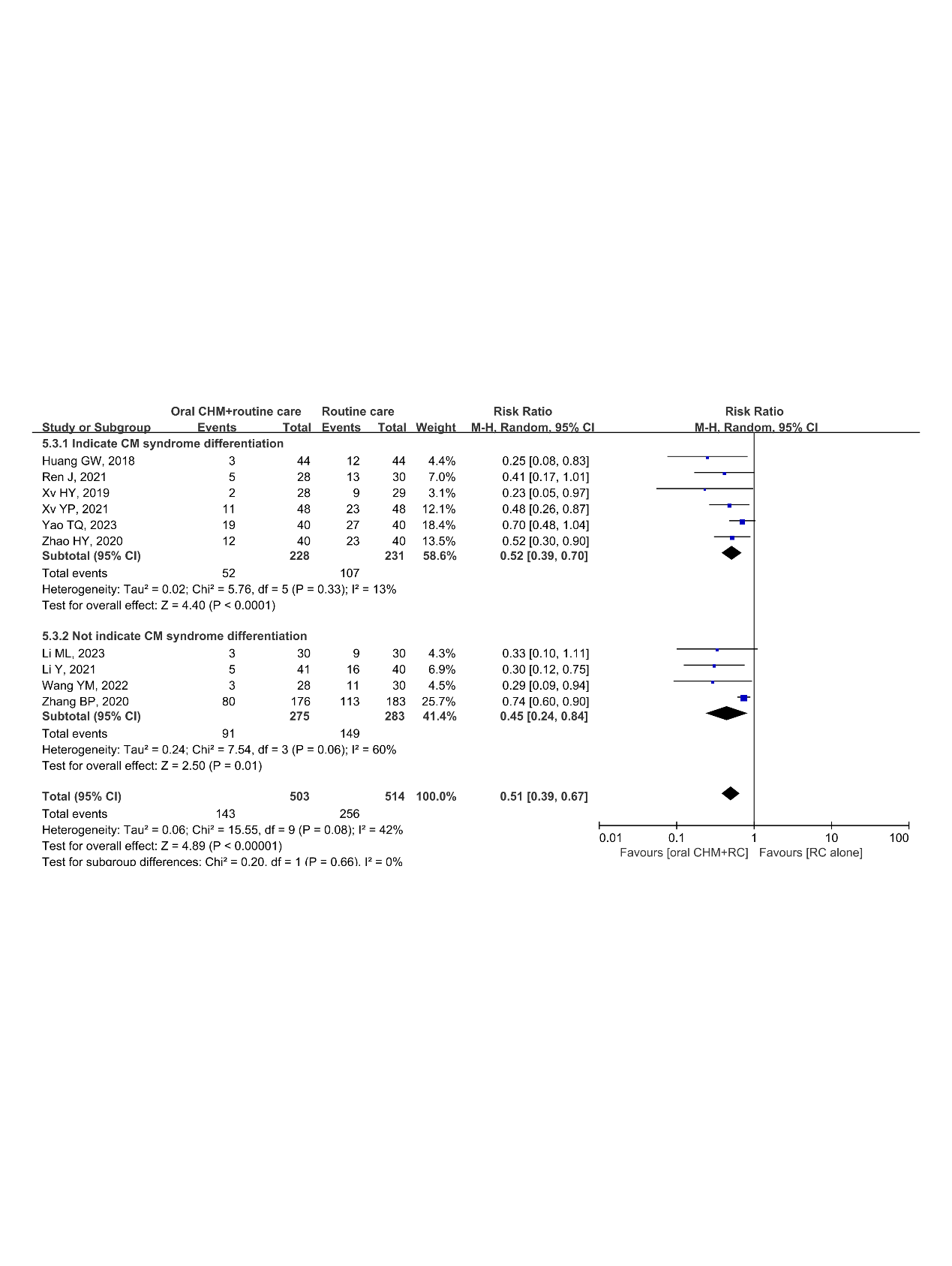


Figure S3.3 Forest plot of CRA recurrence rate at 12 months for oral CHM plus RC versus RC alone for *Tiao chang xiao liu* formula in random-effect model analysis


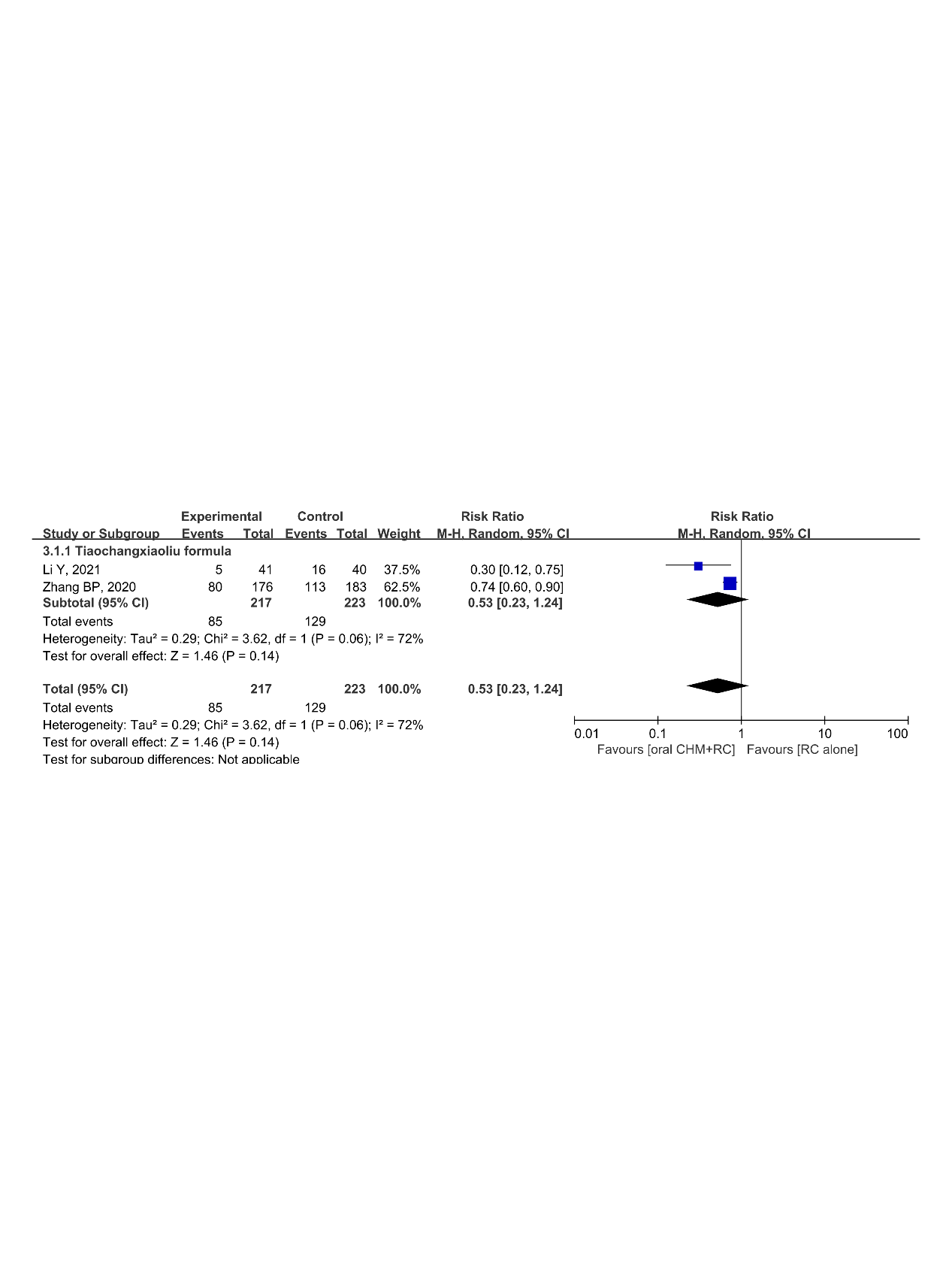


Figure S3.4 Forest plot of CRA recurrence rate at 12 months for oral CHM plus RC versus RC alone for *Tiao chang xiao liu* formula in fixed-effect model analysis


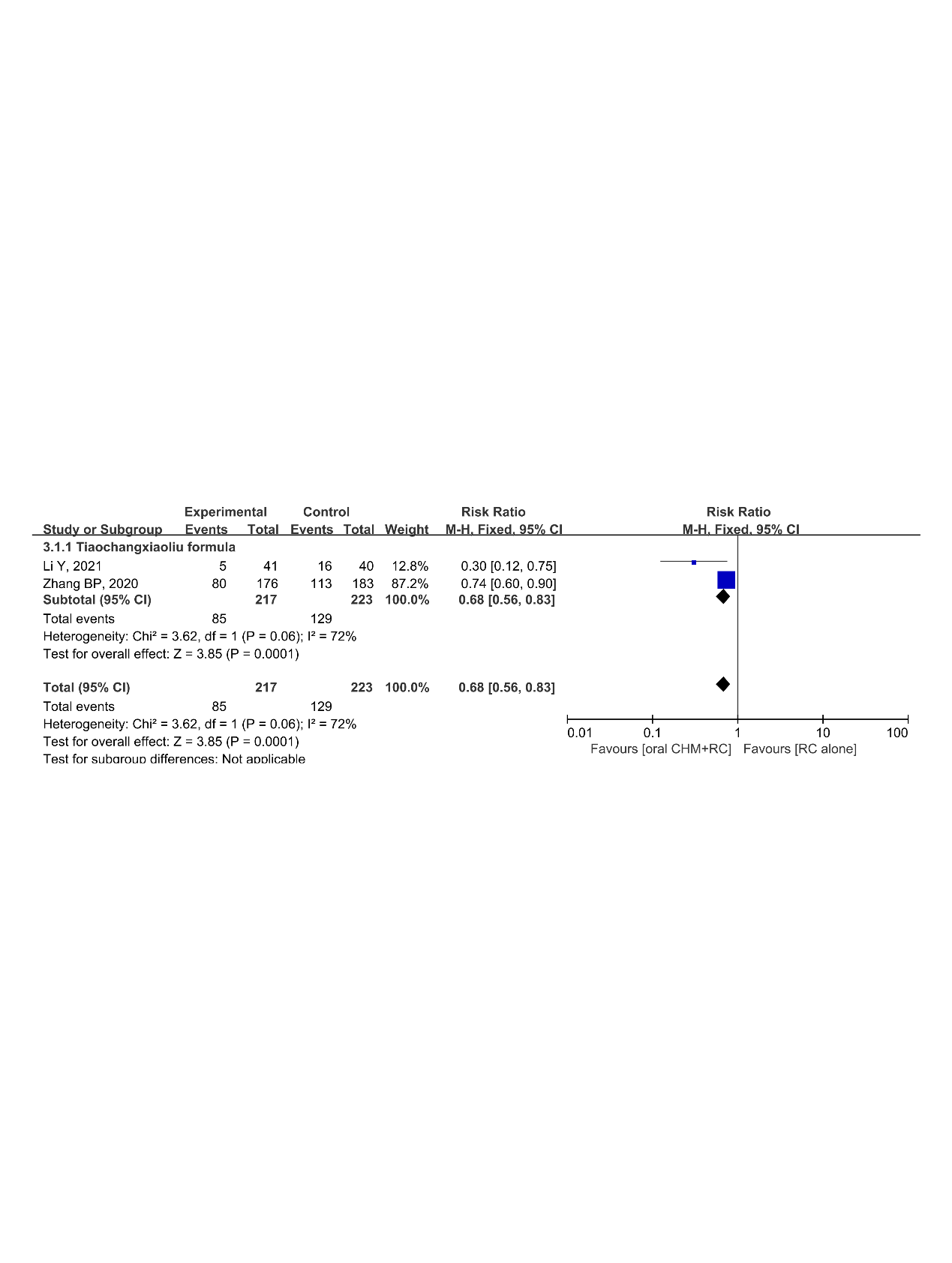


Figure S3.5 Forest plot of CRA recurrence rate at 12 months for oral CHM plus RC versus RC alone for CHMs including or not including *Si jun zi* decoction


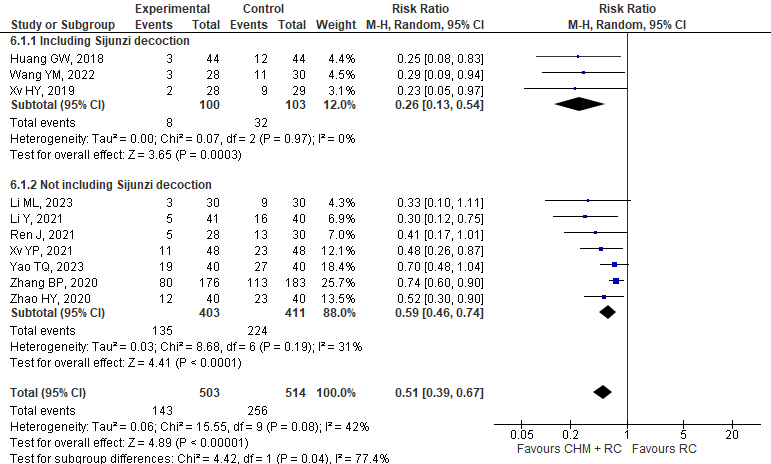


Figure S3.6 Forest plot of CRA recurrence rate at six months for oral CHM plus RC versus RC alone with groups for treatment duration


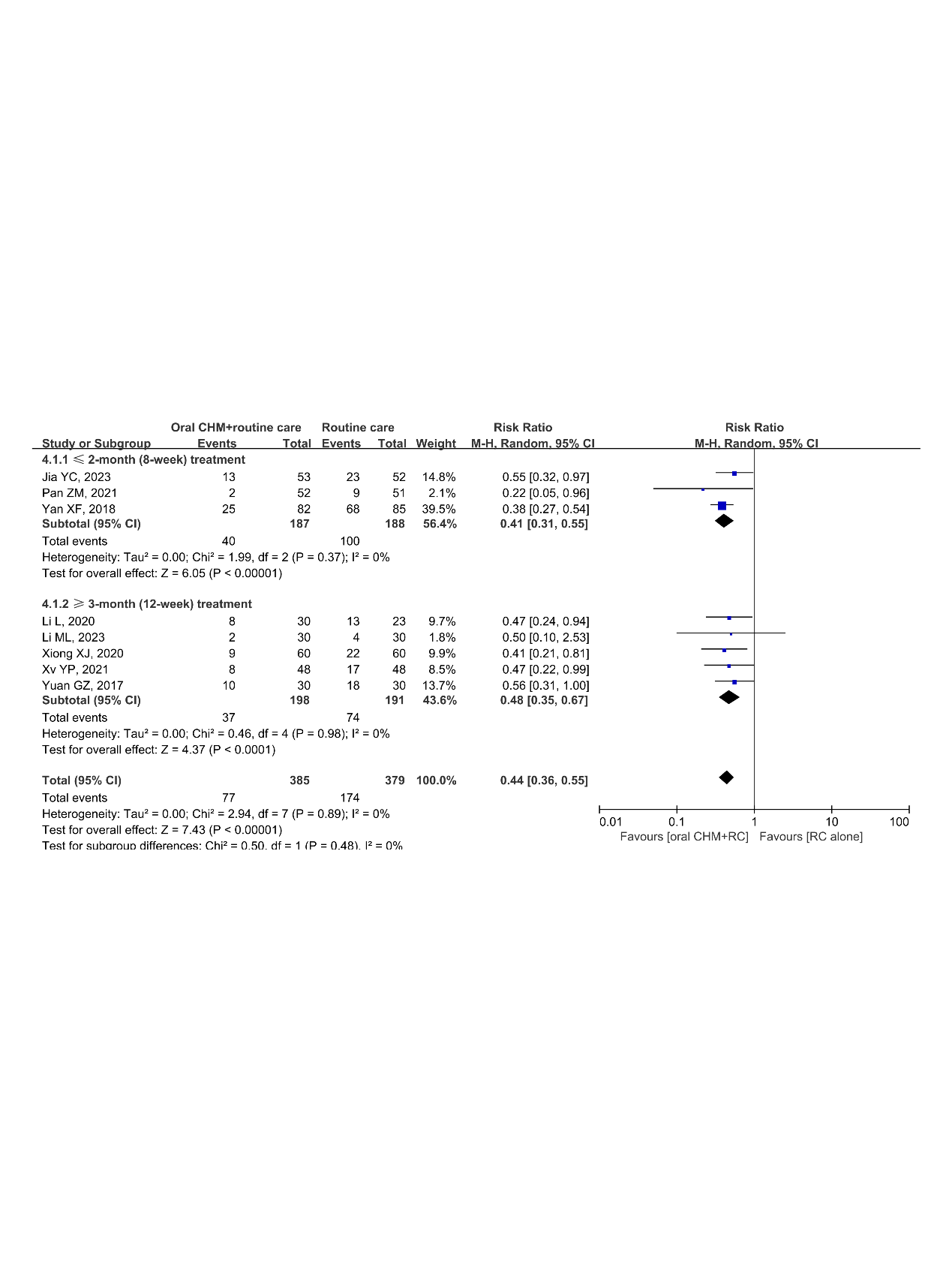


Figure S3.7 Forest plot of CRA recurrence rate at six months for oral CHM plus RC versus RC alone with groups for use of CM syndrome differentiation


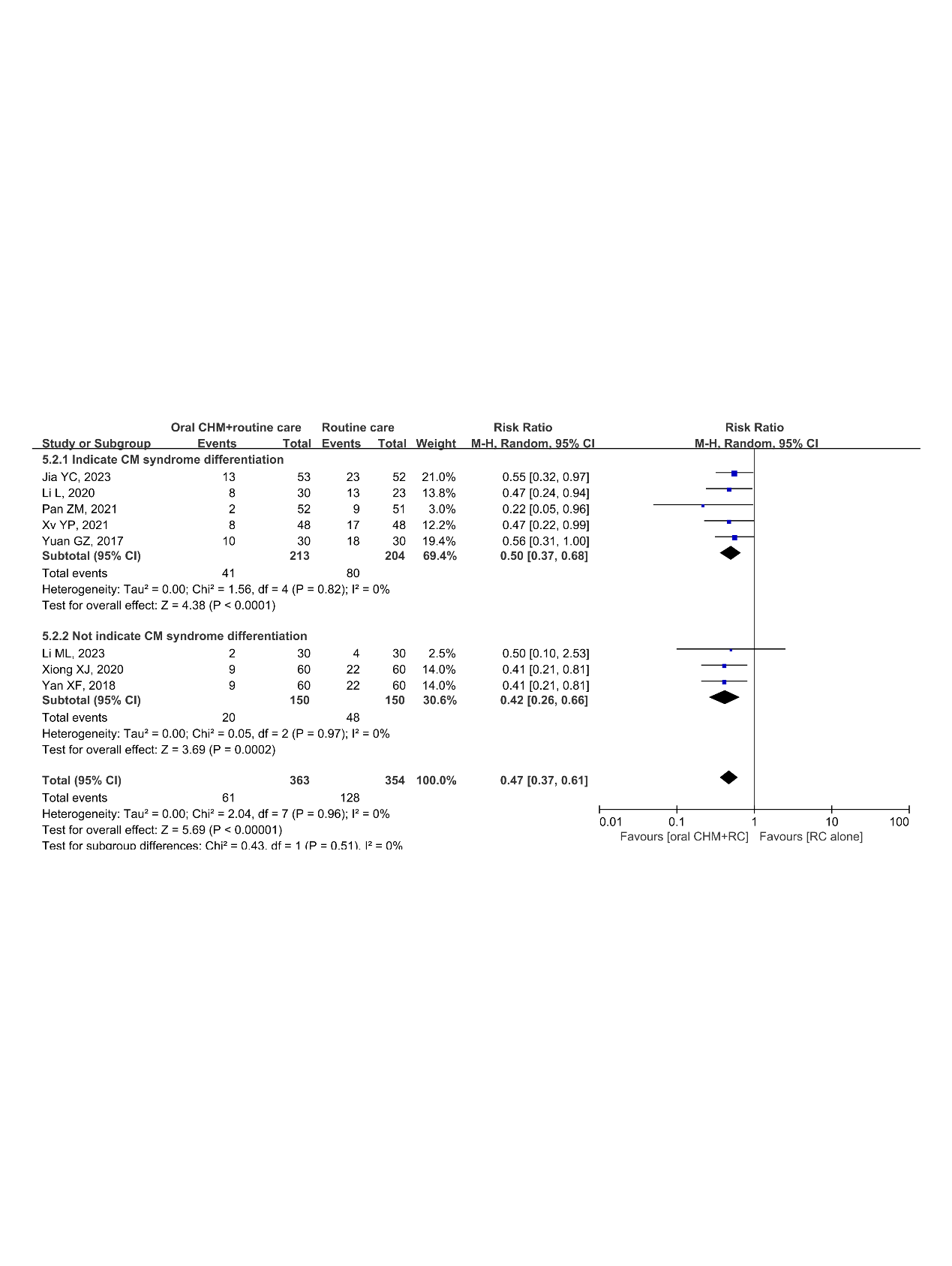


Figure S3.8 Forest plot of CRA recurrence rate at six months for oral CHM plus RC versus RC for CHMs including or not including *Si jun zi* decoction

**
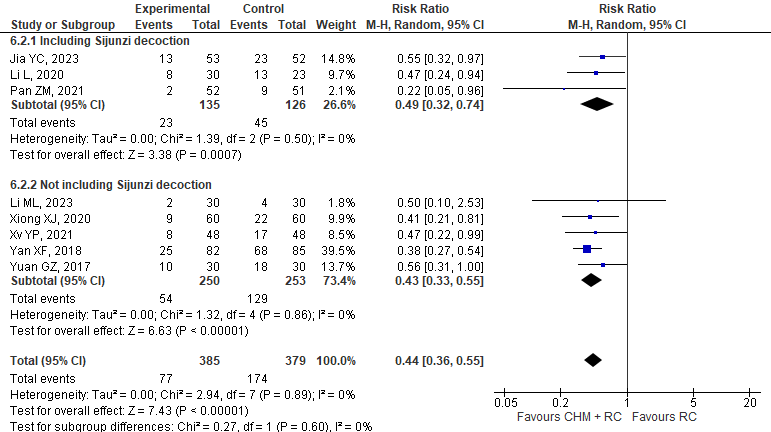
**

# 4 Sensitivity analyses

Figure S4.1 Using the leave-one-study method for sensitivity analysis of the comparison of the effectiveness of oral CHM plus RC versus RC at 12 months after polypectomy


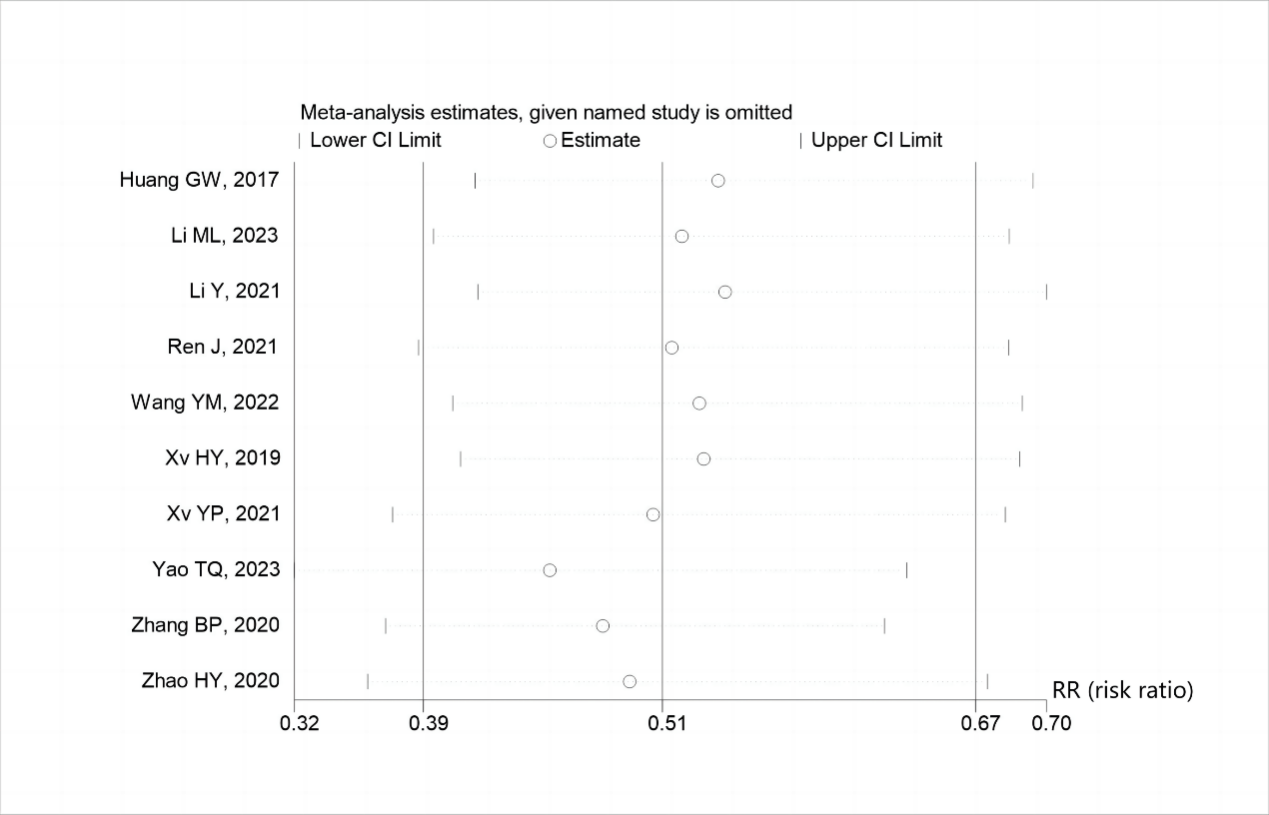


Table S4.1 Results of sensitivity analysis of the comparison of the effectiveness of oral CHM plus RC versus RC at 12 months after polypectomy

| Study omitted | RR [95% CI] |
| --- | --- |
| Huang GW, 2017 | 0.54 [0.41, 0.70] |
| Li ML, 2023 | 0.52 [0.39, 0.68] |
| Li Y, 2021 | 0.54 [0.42, 0.70] |
| Ren J, 2021 | 0.51 [0.39, 0.68] |
| Wang YM, 2022 | 0.53 [0.40, 0.69] |
| Xv HY, 2019 | 0.53 [0.41, 0.69] |
| Xv YP, 2021 | 0.50 [0.37, 0.68] |
| Yao TQ, 2023 | 0.45 [0.32, 0.63] |
| Zhang BP, 2020 | 0.48 [0.37, 0.62] |
| Zhao HY, 2020 | 0.49 [0.36, 0.67] |
| Combined | 0.51 [0.39, 0.67] |

Figure S4.2 Using the leave-one-study method for sensitivity analysis of the comparison of the effectiveness of oral CHM plus RC versus RC at six months after polypectomy


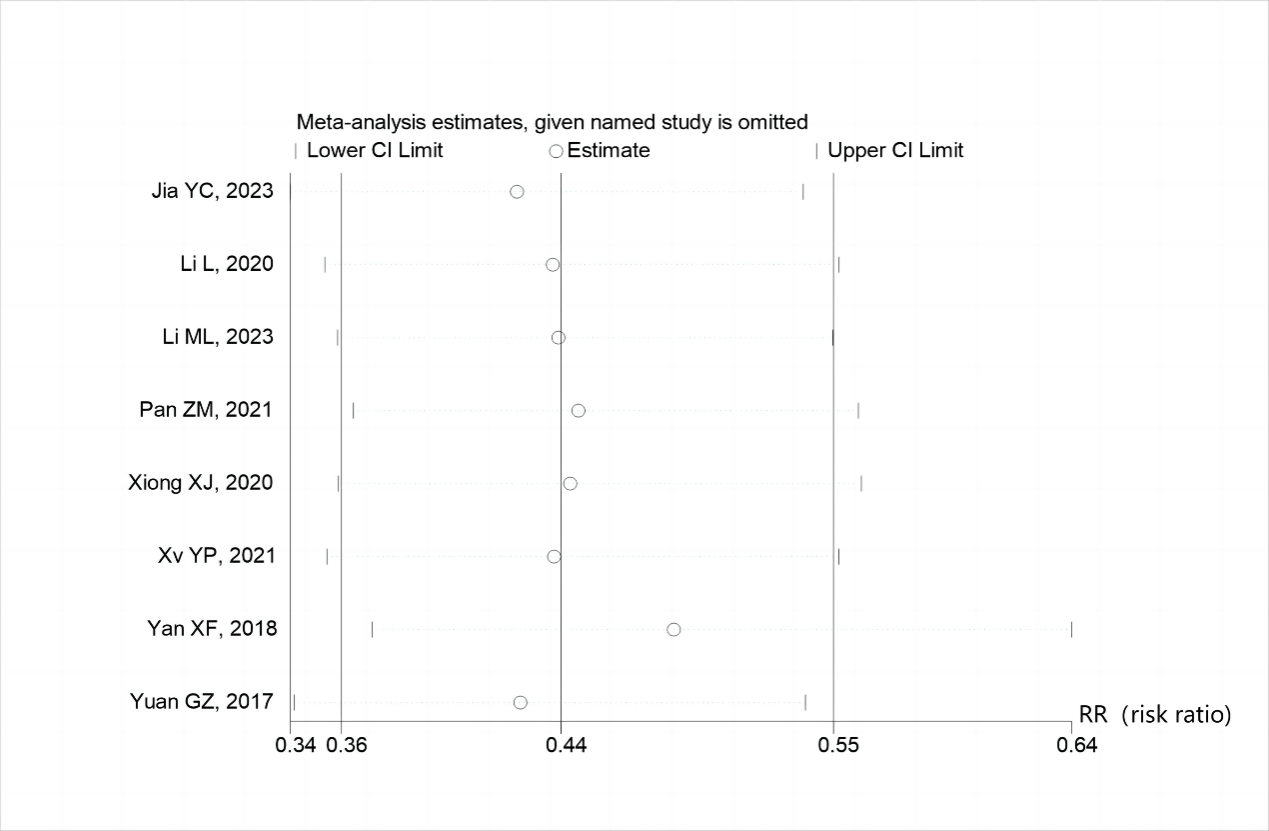


Table S4.2 Results of sensitivity analysis of the comparison of the effectiveness of oral CHM plus RC versus RC at six months after polypectomy

| Study omitted | RR [95% CI] |
| --- | --- |
| Jia YC, 2023 | 0.42 [0.33, 0.54] |
| Li L, 2020 | 0.44 [0.35, 0.55] |
| Li ML, 2023 | 0.44 [0.35, 0.55] |
| Pan ZM, 2021 | 0.45 [0.36, 0.56] |
| Xiong XJ, 2020 | 0.44 [0.35, 0.56] |
| Xv YP, 2021 | 0.44 [0.35, 0.55] |
| Yan XF, 2018 | 0.49 [0.37, 0.64] |
| Yuan GZ, 2017 | 0.42 [0.34, 0.54] |
| Combined | 0.44 [0.36, 0.55] |

# 5 Forest plot of CRA recurrence rate at three months after polypectomy

Figure S5 Forest plot of CRA recurrence rate at three months after polypectomy


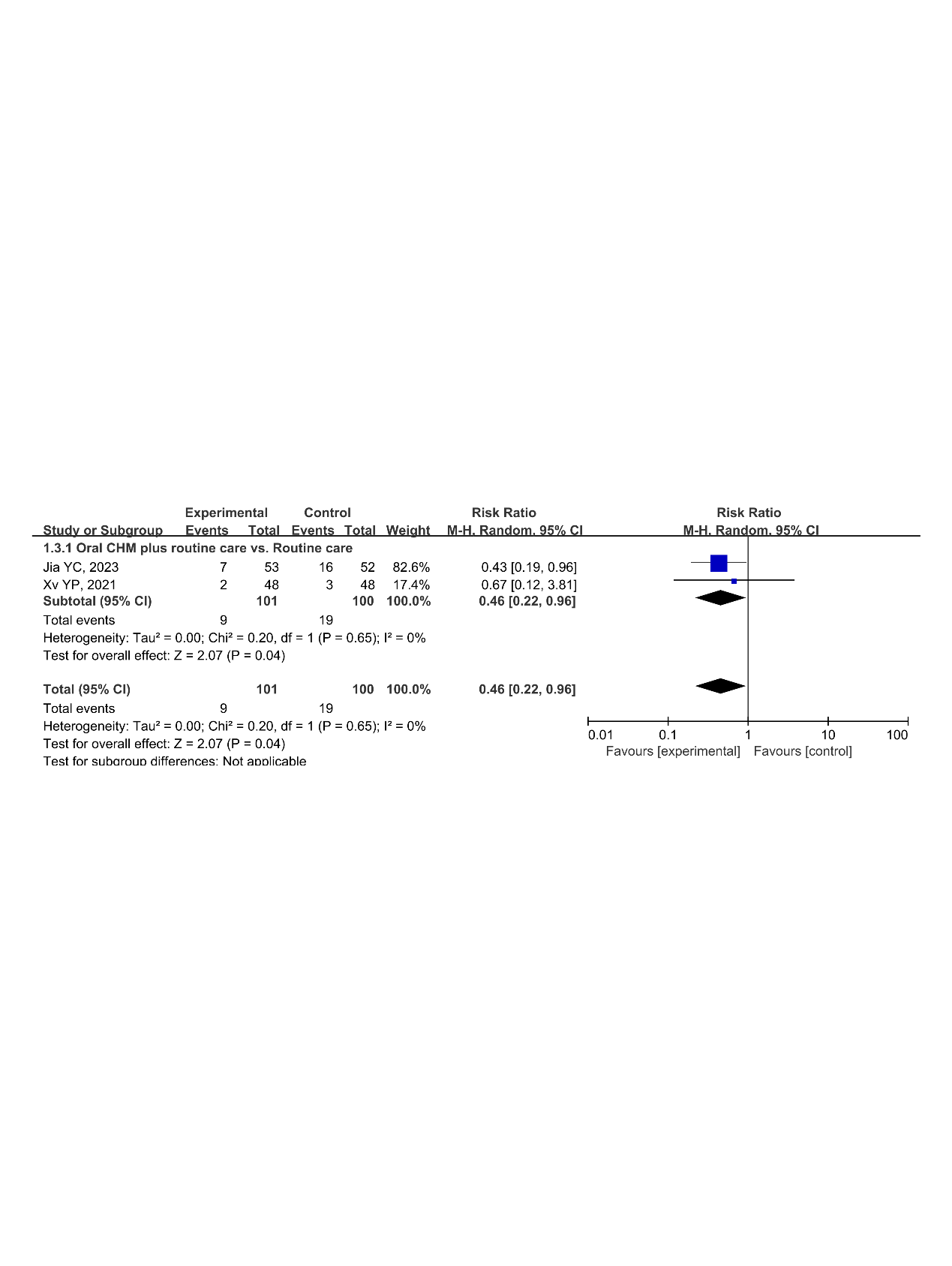


# 6 Forest plot of CRA recurrence rate during 2-year follow-up after polypectomy

Figure S6 Forest plot of CRA recurrence rate during 2-year follow-up after polypectomy


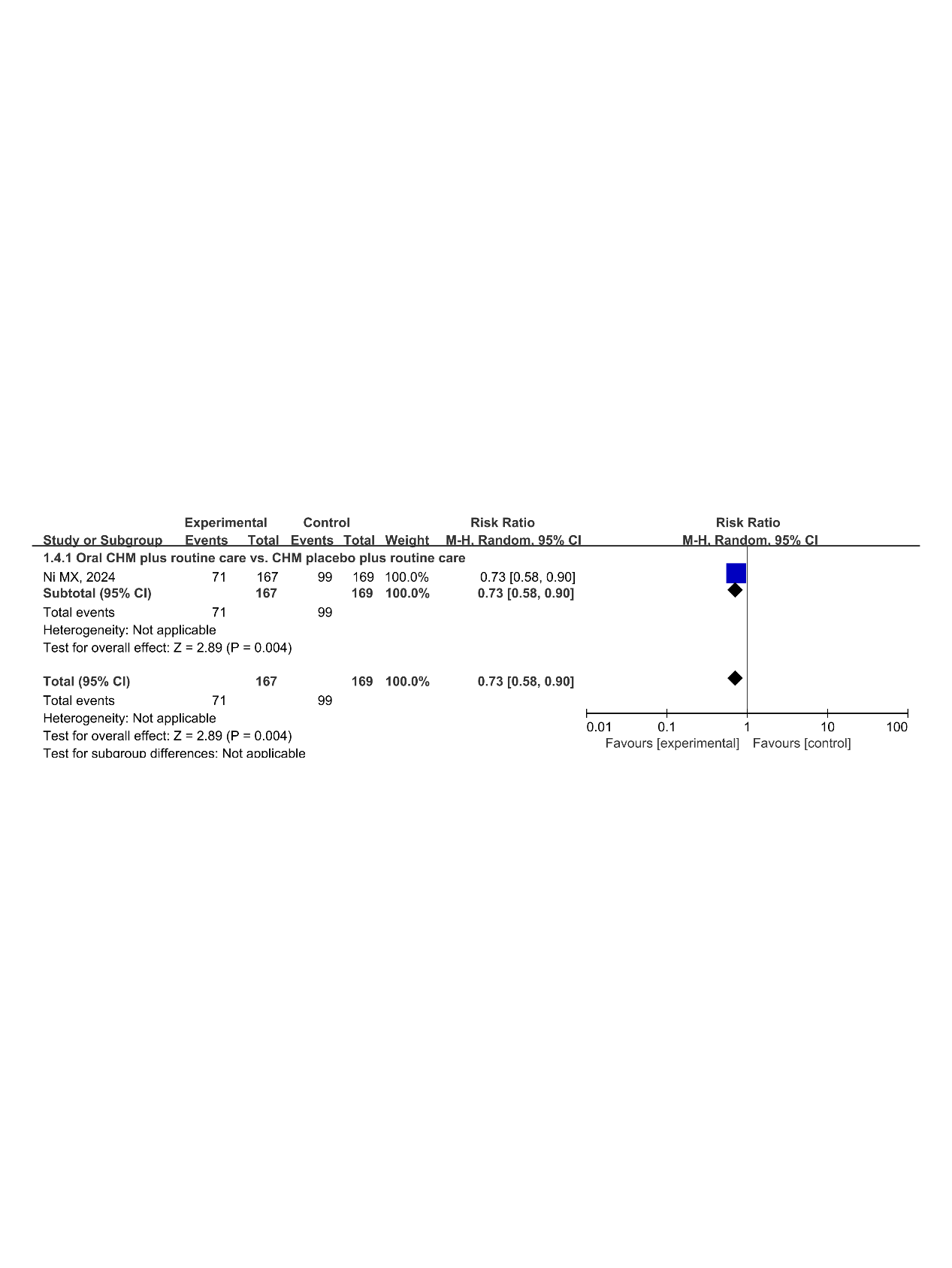


# 7 Forest plot of AEs during the follow-up period

Figure S7 Forest plot of AEs during the follow-up period (3 months to 2 years)


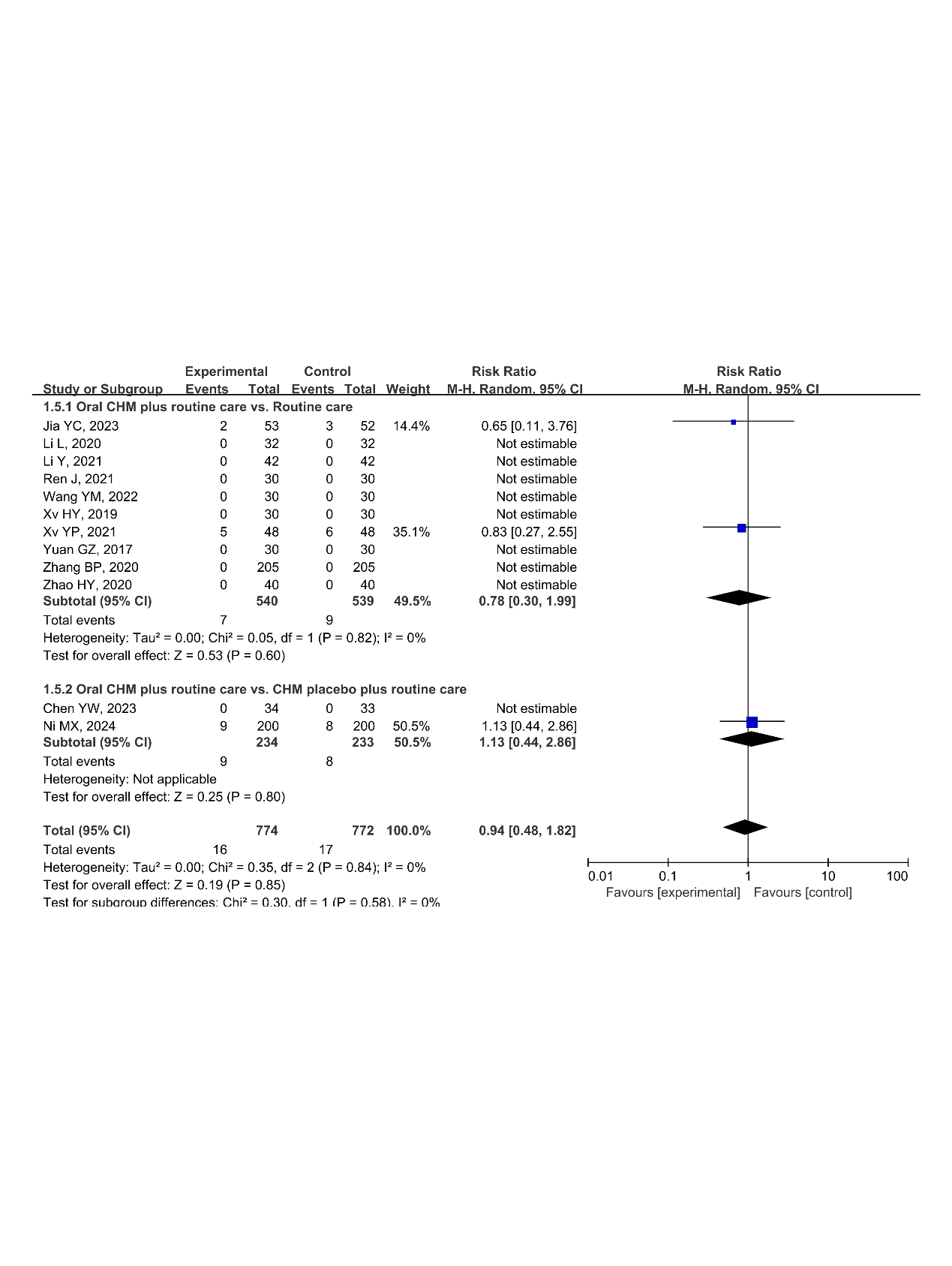


Note: Most studies reported zero AEs in each group.

# 8 Funnel plots

Figure S8 Funnel plot of CRA recurrence rate for oral CHM plus RC compared to RC at 12 months after polypectomy


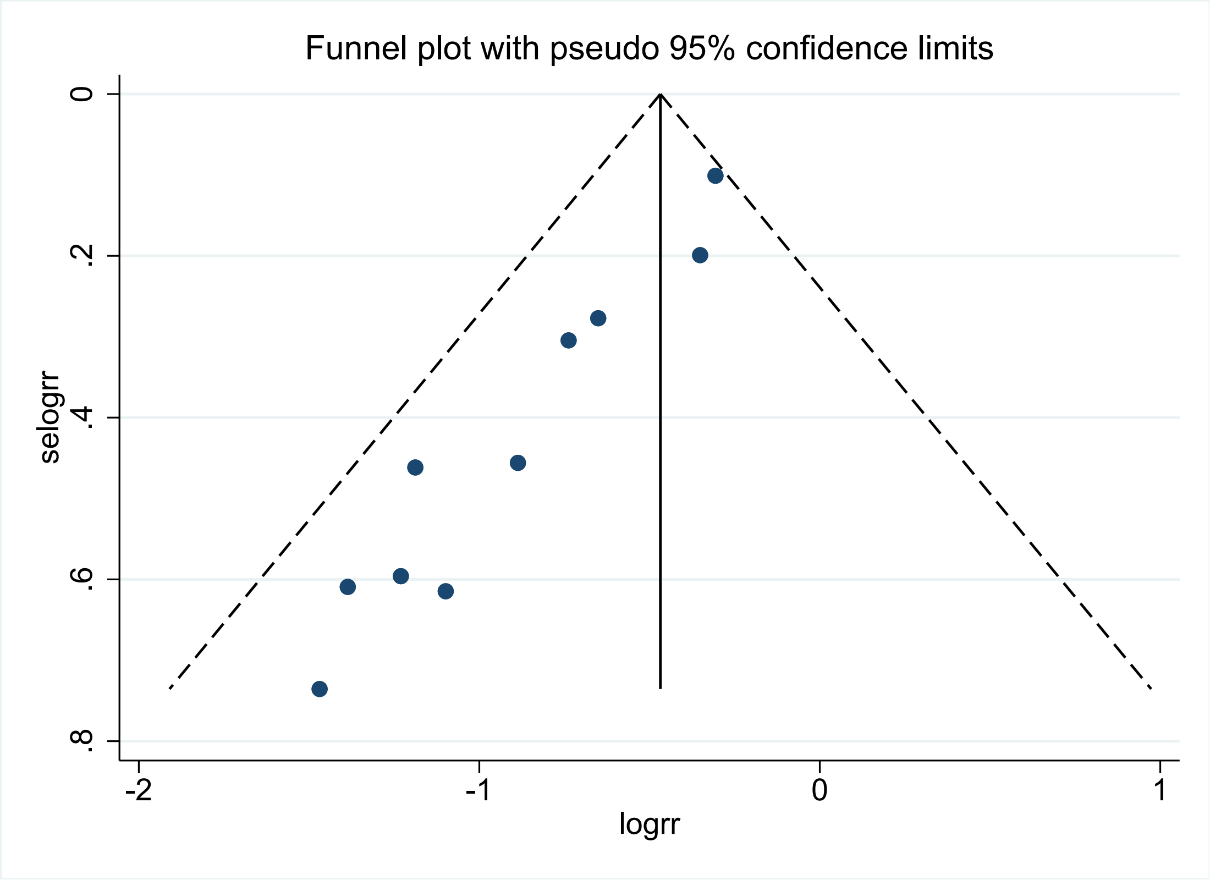


# 9 Egger’s test

Figure S9.1 Egger’s test of CRA recurrence rate in the comparison of oral CHM plus RC and RC at 12 months after polypectomy


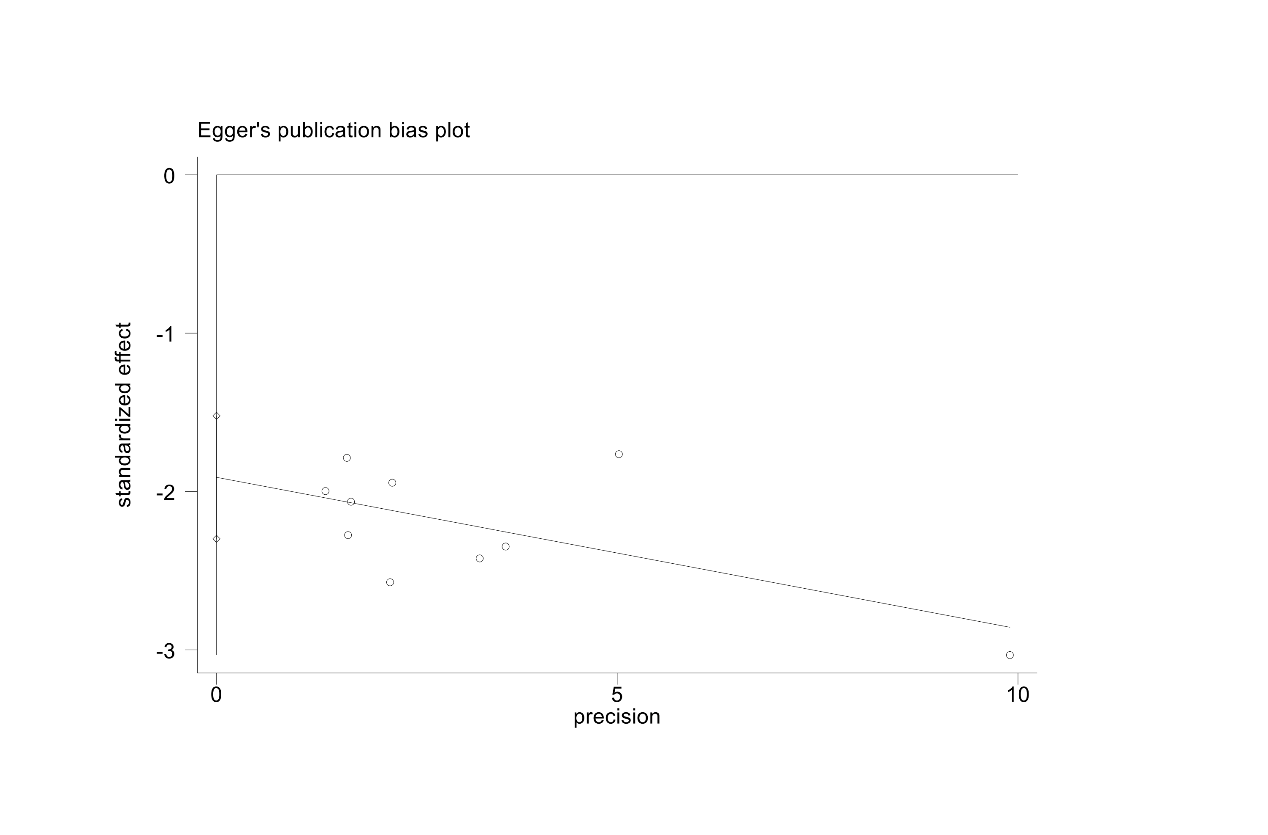


Table S9.1 Results for Egger’s test

| t | 95% CI | p |
| --- | --- | --- |
| -11.34 | -2.30, -1.52 | 0.000 |

# 10 Search strategy

Appendix S10 Search strategy

Pubmed

| Search number | Query |
| --- | --- |
| 1 | Intestinal Polyps[MeSH Terms] |
| 2 | Adenomatous Polyp |
| 3 | colorectal adenoma |
| 4 | colorectal polyp |
| 5 | colorectum adenoma |
| 6 | colorectum polyp |
| 7 | colon adenoma |
| 8 | colon polyp |
| 9 | colonic adenoma |
| 10 | colonic polyp |
| 11 | rectum adenoma |
| 12 | rectum polyp |
| 13 | rectal adenoma |
| 14 | rectal polyp |
| 15 | (((((((((((((Intestinal Polyps[MeSH Terms]) OR (Adenomatous Polyp)) OR (colorectal adenoma)) OR (colorectal polyp)) OR (colorectum adenoma)) OR (colorectum polyp)) OR (colon adenoma)) OR (colon polyp)) OR (colonic adenoma)) OR (colonic polyp)) OR (rectum adenoma)) OR (rectum polyp)) OR (rectal adenoma)) OR (rectal polyp) |
| 16 | Traditional Chinese Medicine |
| 17 | Chinese Traditional Medicine |
| 18 | Chinese Herbal Drugs |
| 19 | Chinese Drugs, Plant |
| 20 | Medicine, Traditional |
| 21 | Ethnopharmacology |
| 22 | Ethnomedicine |
| 23 | Ethnobotany |
| 24 | Medicine, Kampo |
| 25 | TCM |
| 26 | Medicine, Ayurvedic |
| 27 | Phytotherapy |
| 28 | Herbology |
| 29 | Plants, Medicinal |
| 30 | Plant Preparation |
| 31 | Plant Extract |
| 32 | Plants, Medicine |
| 33 | Materia Medica |
| 34 | Single Prescription |
| 35 | Chinese Medicine Herb |
| 36 | Herbal Medicine |
| 37 | Herbs |
| 38 | kampo |
| 39 | ((((((((((((((((((((((Traditional Chinese Medicine) OR (Chinese Traditional Medicine)) OR (Chinese Herbal Drugs)) OR (Chinese Drugs, Plant)) OR (Medicine, Traditional)) OR (Ethnopharmacology)) OR (Ethnomedicine)) OR (Ethnobotany)) OR (Medicine, Kampo)) OR (TCM)) OR (Medicine, Ayurvedic)) OR (Phytotherapy)) OR (Herbology)) OR (Plants, Medicinal)) OR (Plant Preparation)) OR (Plant Extract)) OR (Plants, Medicine)) OR (Materia Medica)) OR (Single Prescription)) OR (Chinese Medicine Herb)) OR (Herbal Medicine)) OR (Herbs)) OR (kampo) |
| 40 | randomized controlled trial[Publication Type] |
| 41 | controlled clinical trial[Publication Type] |
| 42 | randomized[Title/Abstract] |
| 43 | placebo[Title/Abstract] |
| 44 | drug therapy[MeSH Subheading] |
| 45 | randomly[Title/Abstract] |
| 46 | trial[Title/Abstract] |
| 47 | groups[Title/Abstract] |
| 48 | (((((((randomized controlled trial[Publication Type]) OR (controlled clinical trial[Publication Type])) OR (randomized[Title/Abstract])) OR (placebo[Title/Abstract])) OR (drug therapy[MeSH Subheading])) OR (randomly[Title/Abstract])) OR (trial[Title/Abstract])) OR (groups[Title/Abstract]) |
| 49 | (((((((((((((((Intestinal Polyps[MeSH Terms]) OR (Adenomatous Polyp)) OR (colorectal adenoma)) OR (colorectal polyp)) OR (colorectum adenoma)) OR (colorectum polyp)) OR (colon adenoma)) OR (colon polyp)) OR (colonic adenoma)) OR (colonic polyp)) OR (rectum adenoma)) OR (rectum polyp)) OR (rectal adenoma)) OR (rectal polyp)) AND (((((((((((((((((((((((Traditional Chinese Medicine) OR (Chinese Traditional Medicine)) OR (Chinese Herbal Drugs)) OR (Chinese Drugs, Plant)) OR (Medicine, Traditional)) OR (Ethnopharmacology)) OR (Ethnomedicine)) OR (Ethnobotany)) OR (Medicine, Kampo)) OR (TCM)) OR (Medicine, Ayurvedic)) OR (Phytotherapy)) OR (Herbology)) OR (Plants, Medicinal)) OR (Plant Preparation)) OR (Plant Extract)) OR (Plants, Medicine)) OR (Materia Medica)) OR (Single Prescription)) OR (Chinese Medicine Herb)) OR (Herbal Medicine)) OR (Herbs)) OR (kampo))) AND ((((((((randomized controlled trial[Publication Type]) OR (controlled clinical trial[Publication Type])) OR (randomized[Title/Abstract])) OR (placebo[Title/Abstract])) OR (drug therapy[MeSH Subheading])) OR (randomly[Title/Abstract])) OR (trial[Title/Abstract])) OR (groups[Title/Abstract])) |

Cochrane

| Search number | Query |
| --- | --- |
| #1 | MeSH descriptor: [Intestinal Polyps] explode all trees |
| #2 | (Traditional Chinese Medicine OR Chinese Traditional Medicine OR Chinese Herbal Drugs OR Chinese Drugs, Plant OR Medicine, Traditional OR Ethnopharmacology OR Ethnomedicine OR Ethnobotany OR Medicine, Kampo OR Kampo OR TCM OR Medicine, Ayurvedic OR Phytotherapy OR Herbology OR Plants, Medicinal OR Plant Preparation OR Plant Extract OR Plants, Medicine OR Materia Medica OR Single Prescription OR Chinese Medicine Herb OR Herbal Medicine OR Herbs) |
| #3 | MeSH descriptor: [] explode all trees and with qualifier(s): [drug therapy - DT] |
| #4 | (randomized controlled trial):pt |
| #5 | (controlled clinical trial):pt |
| #6 | (randomized):ti,ab,kw |
| #7 | (placebo):ti,ab,kw |
| #8 | (randomly):ti,ab,kw |
| #9 | (trial):ti,ab,kw |
| #10 | (groups):ti,ab,kw |
| #11 | #3 OR #4 OR #5 OR #6 OR #7 OR #8 OR #9 OR #10 |
| #12 | (Adenomatous Polyp) |
| #13 | (colorectal adenoma) |
| #14 | (colorectal polyp) |
| #15 | (colorectum adenoma) |
| #16 | (colorectum polyp) |
| #17 | (colon adenoma) |
| #18 | (colon polyp) |
| #19 | (colonic adenoma) |
| #20 | (colonic polyp) |
| #21 | (rectum adenoma) |
| #22 | (rectum polyp) |
| #23 | (rectal adenoma) |
| #24 | (rectal polyp) |
| #25 | #1 OR #12 OR #13 OR #14 OR #15 OR #16 OR #17 OR #18 OR #19 OR #20 OR #21 OR #22 OR #23 OR #24 |
| #26 | #25 AND #2 AND #11 |

Embase

| Search number | Query |
| --- | --- |
| #1 | ‘intestine polyp’/exp/mj |
| #2 | adenomatous AND polyp |
| #3 | colorectal AND adenoma |
| #4 | colorectal AND polyp |
| #5 | colorectum AND adenoma |
| #6 | colorectum AND polyp |
| #7 | colon AND adenoma |
| #8 | colon AND polyp |
| #9 | colonic AND adenoma |
| #10 | colonic AND polyp |
| #11 | rectum AND adenoma |
| #12 | rectum AND polyp |
| #13 | rectal AND adenoma |
| #14 | rectal AND polyp |
| #15 | #1 OR #2 OR #3 OR #4 OR #5 OR #6 OR #7 OR #8 OR #9 OR #10 OR #11 OR #12 OR #13 OR #14 |
| #16 | traditional AND chinese AND medicine |
| #17 | chinese AND traditional AND medicine |
| #18 | chinese AND herbal AND drugs |
| #19 | chinese AND drugs, AND plant |
| #20 | medicine AND traditional |
| #21 | ethnopharmacology |
| #22 | ethnomedicine |
| #23 | ethnobotany |
| #24 | medicine AND kampo |
| #25 | kampo |
| #26 | tcm |
| #27 | medicine AND ayurvedic |
| #28 | phytotherapy |
| #29 | herbology |
| #30 | plants AND medicinal |
| #31 | plant AND preparation |
| #32 | plant AND extract |
| #33 | plants AND medicine |
| #34 | materia AND medica |
| #35 | single AND prescription |
| #36 | chinese AND medicine AND herb |
| #37 | herbal AND medicine |
| #38 | herbs |
| #39 | ‘chinese medicine’ |
| #40 | #16 OR #17 OR #18 OR #19 OR #20 OR #21 OR #22 OR #23 OR #24 OR #25 OR #26 OR #27 OR #28 OR #29 OR #30 OR #31 OR #32 OR #33 OR #34 OR #35 OR #36 OR #37 OR #38 OR #39 |
| #41 | ‘randomized controlled trial’:it |
| #42 | ‘controlled clinical trial’:it |
| #43 | randomized:ab,ti |
| #44 | placebo:ab,ti |
| #45 | randomly:ab,ti |
| #46 | trial:ab,ti |
| #47 | groups:ab,ti |
| #48 | ‘drug therapy’:lnk |
| #49 | #41 OR #42 OR #43 OR #44 OR #45 OR #46 OR #47 OR #48 |
| #50 | #15 AND #40 AND #49 |

AMED

| Search number | Query |
| --- | --- |
| 1 | (Intestinal Polyps OR Adenomatous Polyp OR colorect* adenoma OR colorect* polyp OR colon* adenoma OR colon* polyp OR rect* adenoma OR rect* polyp) AND (Traditional Chinese Medicine OR Chinese Traditional Medicine OR Chinese Herbal Drugs OR Chinese Drugs, Plant OR Medicine, Traditional OR Ethnopharmacology OR Ethnomedicine OR Ethnobotany OR Medicine, Kampo OR Kampo OR TCM OR Medicine, Ayurvedic OR Phytotherapy OR Herbology OR Plants, Medicinal OR Plant Preparation OR Plant Extract OR Plants, Medicine OR Materia Medica OR Single Prescription OR Chinese Medicine Herb OR Herbal Medicine OR Herbs) AND (randomized controlled trial OR controlled clinical trial OR randomized OR placebo OR drug therapy OR randomly OR trial OR groups) |

CINAHL

| Search number | Query |
| --- | --- |
| 1 | TX Traditional Chinese Medicine OR Chinese Traditional Medicine OR Chinese Herbal Drugs OR Chinese Drugs, Plant OR Medicine, Traditional OR Ethnopharmacology OR Ethnomedicine OR Ethnobotany OR Medicine, Kampo OR Kampo OR TCM OR Medicine, Ayurvedic OR Phytotherapy OR Herbology OR Plants, Medicinal OR Plant Preparation OR Plant Extract OR Plants, Medicine OR Materia Medica OR Single Prescription OR Chinese Medicine Herb OR Herbal Medicine OR Herbs |
| 2 | TX Intestinal Polyps OR Adenomatous Polyp OR colorect* adenoma OR colorect* polyp OR colon* adenoma OR colon* polyp OR rect* adenoma OR rect* polyp |
| 3 | PT (“randomized controlled trial” OR “controlled clinical trial”) OR drug therapy OR TI (“randomized” OR “placebo” OR “randomly” OR “trial” OR “groups”) OR AB (“randomized” OR “placebo” OR “randomly” OR “trial” OR “groups”) |
| 4 | 1 AND 2 AND 3 |

CNKI

| Search number | Query |
| --- | --- |
| 1 | (SU%腺瘤性息肉 OR SU%结直肠腺瘤 OR SU%结直肠息肉 OR SU%结肠腺瘤 OR SU%结肠息肉 OR SU%直肠腺瘤 OR SU%直肠息肉 OR SU%大肠腺瘤 OR SU%大肠息肉 OR SU%肠腺瘤 OR SU%肠息肉) AND (FT=中医 OR FT=中西医 OR FT=辨病 OR FT=辨证 OR FT=传统医学 OR FT=传统治疗 OR FT=传统疗法 OR FT=替代医学 OR FT=替代治疗 OR FT=草药 OR FT=中草药 OR FT=中药 OR FT=中西药 OR FT=传统医药 OR FT=中成药 OR FT=治则 OR FT=外敷 OR FT=灌肠) AND (FT=随机 OR FT=安慰剂 OR FT=对照) |

CBM

| Search number | Query |
| --- | --- |
| #1 | “腺瘤性息肉”[加权:扩展] OR “结直肠腺瘤”[全部字段:智能] OR “结直肠息肉”[全部字段:智能] OR “结肠腺瘤”[常用字段:智能] OR “直肠息肉”[常用字段:智能] OR “大肠腺瘤”[常用字段:智能] OR “大肠息肉”[常用字段:智能] OR “肠腺瘤"[常用字段:智能] OR “肠息肉”[常用字段:智能] OR “结肠息肉”[常用字段:智能] OR “直肠腺瘤”[常用字段:智能] |
| #2 | 中医 OR 中西医 OR 辨病 OR 辨证 OR 传统医学 OR 传统治疗 OR 传统疗法 OR 替代医学 OR 替代治疗 OR 草药 OR 中草药 OR 中药 OR 中西药 OR 传统医药 OR 中成药 OR 治则 OR 外敷 OR 灌肠 |
| #3 | 随机 OR 安慰剂 OR 对照 |
| #4 | (#3) AND (#2) AND (#1) |

CQVIP

| Search number | Query |
| --- | --- |
| 1 | U=(中医 OR 中西医 OR 辨病 OR 辨证 OR 传统医学 OR 传统治疗 OR 传统疗法 OR 替代医学 OR 替代治疗 OR 草药 OR 中草药 OR 中药 OR 中西药 OR 传统医药 OR 中成药 OR 治则 OR 外敷 OR 灌肠) AND U=(随机 OR 安慰剂 OR 对照) AND M=(腺瘤性息肉 OR 结直肠腺瘤 OR 结直肠息肉 OR 结肠腺瘤 OR 结肠息肉 OR 直肠腺瘤 OR 直肠息肉 OR 大肠腺瘤 OR 大肠息肉 OR 肠腺瘤 OR 肠息肉) |

Wanfang

| Search number | Query |
| --- | --- |
| 1 | (中医 OR 中西医 OR 辨病 OR 辨证 OR 传统医学 OR 传统治疗 OR 传统疗法 OR 替代医学 OR 替代治疗 OR 草药 OR 中草药 OR 中药 OR 中西药 OR 传统医药 OR 中成药 OR 治则 OR 外敷 OR 灌肠) AND (随机 OR 安慰剂 OR 对照) AND (腺瘤性息肉 OR 结直肠腺瘤 OR 结直肠息肉 OR 结肠腺瘤 OR 结肠息肉 OR 直肠腺瘤 OR 直肠息肉 OR 大肠腺瘤 OR 大肠息肉 OR 肠腺瘤 OR 肠息肉) |

# 11 Information of AEs

Table S11.1 Information of AEs in comparison of oral CHM plus RC and RC alone

| AEs | Frequency in oral CHM plus RC group | Frequency in RC group |
| --- | --- | --- |
| Vomiting | 2 | 1 |
| Skin rash | 2 | 3 |
| Nausea | 1 | 2 |
| Abdominal distension | 1 | 2 |
| Dizziness | 1 | 1 |
| All AEs | 7 | 9 |

Note: AEs: adverse events; CHM: Chinese herbal medicine; RC: routine care.

Table S11.2 Information of AEs in comparison of oral CHM plus RC and CHM placebo plus RC

| AEs | Frequency in oral CHM plus RC group | Frequency in CHM placebo plus RC group |
| --- | --- | --- |
| Diarrhea | 4 | 5 |
| Abdominal pain | 1 | 0 |
| Abdominal distention | 1 | 2 |
| Nausea | 1 | 0 |
| Nonspecific dizziness | 1 | 0 |
| Fatigue | 1 | 1 |
| All AEs | 9 | 8 |

Note: AEs: adverse events; CHM: Chinese herbal medicine; RC: routine care.

# 12 GRADE assessments

| Oral CHM plus routine care compared to routine care for CRA recurrence | | | | | | | | | | | |
| --- | --- | --- | --- | --- | --- | --- | --- | --- | --- | --- | --- |
| Certainty assessment | | | | | | | Summary of findings | | | | |
| Participants (studies) Follow-up | Risk of bias | Inconsistency | Indirectness | Imprecision | Publication bias | Overall certainty of evidence | Study event rates (%) | | Relative effect (95% CI) | Anticipated absolute effects | |
|  |  |  |  |  |  |  | With [routine care] | With [Oral CHM plus routine care] |  | Risk with [routine care] | Risk difference with [Oral CHM plus routine care] |
| Recurrence rate of colorectal adenoma at 3 months after polypectomy (follow-up: 3 months; assessed with: Colonoscopy and pathological diagnosis) | | | | | | | | | | | |
| 201 (2 RCTs) | serious^a^ | not serious | not serious^b^ | serious^b^ | none | ⨁⨁◯◯ Low^a,b^ | 19/100 (19.0%) | 9/101 (8.9%) | RR 0.46 (0.22 to 0.96) | 19/100 (19.0%) | 103 fewer per 1,000 (from 148 fewer to 8 fewer) |
| Recurrence rate of colorectal adenoma at 6 months after polypectomy (follow-up: 6 months; assessed with: Colonoscopy and pathological diagnosis) | | | | | | | | | | | |
| 764 (8 RCTs) | serious^c^ | not serious | not serious | not serious | none | ⨁⨁⨁◯ Moderate^c^ | 174/379 (45.9%) | 77/385 (20.0%) | RR 0.44 (0.36 to 0.55) | 174/379 (45.9%) | 257 fewer per 1,000 (from 294 fewer to 207 fewer) |
| Recurrence rate of colorectal adenoma at 12 months after polypectomy (follow-up: 12 months; assessed with: Colonoscopy and pathological diagnosis) | | | | | | | | | | | |
| 1017 (10 RCTs) | serious^d^ | not serious | not serious | not serious | publication bias strongly suspected^e^ | ⨁⨁◯◯ Low^d,e^ | 256/514 (49.8%) | 143/503 (28.4%) | RR 0.51 (0.39 to 0.67) | 256/514 (49.8%) | 244 fewer per 1,000 (from 304 fewer to 164 fewer) |
| Adverse events (assessed with: Clinical assessment) | | | | | | | | | | | |
| 1079 (10 RCTs) | serious^f^ | not serious | not serious | serious^g^ | none | ⨁⨁◯◯ Low^f,g^ | 9/539 (1.7%) | 7/540 (1.3%) | RR 0.78 (0.30 to 1.99) | 9/539 (1.7%) | 4 fewer per 1,000 (from 12 fewer to 17 more) |

Note: CHM, Chinese herbal medicine; CI, confidence interval; CRA: colorectal adenoma; GRADE, Grading of Recommendations Assessment, Development and Evaluation; RR, risk ratio.

a. Downgraded by one level because two studies had some concerns about the risk of bias for the randomisation process and selection of the reported result domains.

b. Downgraded by one level because a small number of participants were included (n = 201) (< 400).

c. Downgrade by one level because seven studies had some concerns about the risk of bias for randomisation process domains, and eight studies had some concerns about selection of the reported result domains.

d. Downgraded by one level because five studies had some concerns about the risk of bias for randomisation process domains, and eight studies had some concerns about the selection of the reported result domains.

e. Downgraded by one level for detected publication bias in funnel plot and Egger’s test.

f. Downgraded by one level because seven studies had some concerns about the risk of bias for randomisation process domains, nine studies had a high risk of bias for measurement of the outcomes domain, and nine studies had some concerns for selection of the reported result domains.

g. Downgraded by one level because 95% CI includes RR of 1.0, showing appreciable benefit and harm.

| [Oral CHM plus routine care] compared to [placebo plus routine care] for [CRA recurrence] | | | | | | | | | | | |
| --- | --- | --- | --- | --- | --- | --- | --- | --- | --- | --- | --- |
| Certainty assessment | | | | | | | Summary of findings | | | | |
| Participants (studies) Follow-up | Risk of bias | Inconsistency | Indirectness | Imprecision | Publication bias | Overall certainty of evidence | Study event rates (%) | | Relative effect (95% CI) | Anticipated absolute effects | |
|  |  |  |  |  |  |  | With [placebo plus routine care] | With [Oral CHM plus routine care] |  | Risk with [placebo plus routine care] | Risk difference with [Oral CHM plus routine care] |
| Recurrence rate of colorectal adenoma at 12 months after polypectomy (follow-up: 12 months; assessed with: Colonoscopy and pathological diagnosis) | | | | | | | | | | | |
| 127 (2 RCTs) | serious^a^ | not serious | not serious | serious^b^ | none | ⨁⨁◯◯ Low^a,b^ | 16/63 (25.4%) | 6/64 (9.4%) | RR 0.39 (0.16 to 0.93) | 16/63 (25.4%) | 155 fewer per 1,000 (from 213 fewer to 18 fewer) |
| Recurrence rate of colorectal adenoma during a 2-year follow-up after polypectomy (follow-up: 2 years; assessed with: Colonoscopy and pathological diagnosis) | | | | | | | | | | | |
| 336 (1 RCT) | not serious | not serious | not serious | serious^c^ | none | ⨁⨁⨁◯ Moderate^c^ | 99/169 (58.6%) | 71/167 (42.5%) | RR 0.73 (0.58 to 0.90) | 99/169 (58.6%) | 158 fewer per 1,000 (from 246 fewer to 59 fewer) |
| Recurrence rate of colorectal adenoma at 6 months after polypectomy (follow-up: 6 months; assessed with: Colonoscopy and pathological diagnosis) | | | | | | | | | | | |
| 60 (1 RCT) | serious^d^ | not serious | not serious | serious^e^ | none | ⨁⨁◯◯ Low^d,e^ | 4/30 (13.3%) | 2/30 (6.7%) | RR 0.50 (0.10 to 2.53) | 4/30 (13.3%) | 67 fewer per 1,000 (from 120 fewer to 204 more) |

Note: CHM, Chinese herbal medicine; CI, confidence interval; CRA: colorectal adenoma; GRADE, Grading of Recommendations Assessment, Development and Evaluation; RR, risk ratio.

a. Downgraded by one level because one study had some concerns for risk of bias for randomisation process and two studies had some concerns for risk of bias for selection of the reported result domains.

b. Downgraded by one level because only two studies with 127 (< 400) participants were included.

c. Downgraded by one level because only one studies with 336 (< 400) participants were included.

d. Downgraded by one level because one study had some concerns for risk of bias for selection of the reported result domains.

e. Downgraded by one level because only one studies with 60 (< 400) participants were included.

| [Oral CHM plus probiotic plus routine care] compared to [probiotic plus routine care] for [CRA recurrence] | | | | | | | | | | | |
| --- | --- | --- | --- | --- | --- | --- | --- | --- | --- | --- | --- |
| Certainty assessment | | | | | | | Summary of findings | | | | |
| Participants (studies) Follow-up | Risk of bias | Inconsistency | Indirectness | Imprecision | Publication bias | Overall certainty of evidence | Study event rates (%) | | Relative effect (95% CI) | Anticipated absolute effects | |
|  |  |  |  |  |  |  | With [probiotic plus routine care] | With [Oral CHM plus probiotic plus routine care] |  | Risk with [probiotic plus routine care] | Risk difference with [Oral CHM plus probiotic plus routine care] |
| Recurrence rate of colorectal adenoma at 12 months after polypectomy (follow-up: 12 months; assessed with: Colonoscopy and pathological diagnosis) | | | | | | | | | | | |
| 81 (1 RCT) | serious^a^ | not serious | not serious | serious^b^ | none | ⨁⨁◯◯ Low^a,b^ | 23/39 (59.0%) | 6/42 (14.3%) | RR 0.24 (0.11 to 0.53) | 23/39 (59.0%) | 448 fewer per 1,000 (from 525 fewer to 277 fewer) |

Note: CHM, Chinese herbal medicine; CI, confidence interval; CRA: colorectal adenoma; GRADE, Grading of Recommendations Assessment, Development and Evaluation; RR, risk ratio.

a. Downgraded by one level because one study had some concerns for risk of bias for randomisation process and some concerns for risk of bias for selection of the reported result domains.

b. Downgraded by one level because only one studies with 81 (< 400) participants were included.
